# Supplementary material for: From static to dynamic: Embracing dynamics in isotopic diet estimation
Source: PLoS One. 2025 Aug 26;20(8):e0330327. doi: 10.1371/journal.pone.0330327 (PMC12380277; doi:10.1371/journal.pone.0330327)
Supplement: S1 Appendix — (DOCX) [file pone.0330327.s001.docx]

Appendix 1: Literature review regarding mixing model use to estimate diet and temporal effects

In exploring the use of mixing models, we sought to assess the extent to which ecosystem dynamics are considered. The corpus for this analysis consisted of 103 peer-reviewed articles published between 2014 and 2024. The starting date, 2014, was chosen because it corresponds to the year of publication of Phillips et al.’s (2014) paper on recommendations for using mixing models in diet estimation. These were selected from Google Scholar using the keywords: "isotopic," "mixing models," "MixSIAR," "ecology," and "diet." Articles were included if they applied mixing models to estimate dietary composition.

For each article, we examined whether the following criteria were addressed: the assumption of a stable or unstable system state, verification of this assumption, multiple measurements of source isotopic signatures over time, and multiple measurements of consumer signatures over time. We also evaluated whether the articles followed the sampling recommendations outlined by Phillips et al. (2014), which include: confirming that the consumer’s isotopic signature lies within the source polygon, averaging source values over an appropriate time window, and accounting for trophic discrimination factors (TDFs). Additionally, we checked whether the consumer's isotopic turnover was mentioned, and whether source values were averaged accordingly. Finally, we recorded whether Phillips et al. (2014) was cited, and categorized the type of data used in each study as either fieldwork, laboratory work, or archaeological research. The last web search was conducted on 30/08/2024.

In total, we analyzed 103 articles, with annual representation ranging from 5 to 18 articles per year. Among these, 6 focused on archaeological research, 7 were laboratory-based studies, and 90 involved fieldwork. In archaeological applications of mixing models, incorporating temporal dynamics is generally not feasible. Similarly, none of the laboratory studies accounted for time-related dynamics. However, fieldwork studies showed greater variability in this regard. A key finding is that only 25% of the articles explicitly assumed either a stable or unstable system. This means that 75% of the articles did not address the equilibrium assumption at all.

Regarding the selected recommendations from Phillips et al. (2014)—namely: ensuring the consumer’s isotopic values fall within the source polygon, averaging source values over a time window, and selecting an appropriate trophic discrimination factor (TDF)—we found the following:

- 5% of articles followed none of the recommendations.
- 56% followed one recommendation, most often the careful selection of a TDF.
- 33% adhered to two recommendations, typically selecting a TDF and checking the source polygon.
- Only 6% followed all three recommendations.

Only 40% of the articles cited Phillips et al. (2014), suggesting that the guidance provided in that paper is not consistently implemented. To illustrate this further, while 87% of the studies carefully selected a TDF, only 18% averaged source values over a time window. This highlights a tendency among researchers to give less importance to temporal dynamics when considering potential biases in diet estimation. Another important aspect of time-related considerations is the consumer’s isotopic turnover rate. Only 26% of the articles mentioned turnover, and just 4% used it to guide sampling within a time frame aligned with the turnover rate. This indicates that turnover receives generally little attention.

Finally, although only 18% of articles averaged source values over time, 43% actually collected multiple source measurements over time—meaning that 22% of studies had the potential to treat their data as time series but did not. Similarly, 54% included multiple consumer measurements over time.

**Table 1: Summary of Criteria from the Review of 103 Articles**

| **Criteria:** | **Number of articles answering the criteria** | **% of articles answering the criteria** |
| --- | --- | --- |
| Field data | 90 | 87% |
| Archeological data | 6 | 6% |
| Lab data | 7 | 6% |
| Equilibrium/no-equilibrium Hypothesis | 26 | 25% |
| Testing Equilibrium/no-equilibrium hypothesis | 26 | 25% |
| Checking if the consumer lies within the source polygon | 30 | 29% |
| Articles presenting several measurement of source signatures over time | 44 | 43% |
| Articles averaging the source signature on a time window | 19 | 18% |
| Articles averaging the source signature on a time window dependent of λ | 4 | 4% |
| Articles presenting several measurement of consumer signatures over time | 56 | 54% |
| Articles choosing carefully the TDF value | 90 | 87% |
| Articles where λ is mentioned | 27 | 26% |
| Articles citing *Phillips et al*.,2014 | 41 | 40% |
| Articles respecting 0 among:  -Consumer within the polygon  -Averaging the sources signatures on a time window  -Choosing carefully the TDF | 5 | 5% |
| Articles respecting 1 among:  -Consumer within the polygon  -Averaging the sources signatures on a time window  -Choosing carefully the TDF | 58 | 56% |
| Articles respecting 2 among:  -Consumer within the polygon  -Averaging the sources signatures on a time window  -Choosing carefully the TDF | 34 | 33% |
| Articles respecting 3 among:  -Consumer within the polygon  -Averaging the sources signatures on a time window  -Choosing carefully the TDF | 6 | 6% |

**List of articles used in this review:**

1. Ambra ID, Graham WM, Carmichael RH, Hernandez Jr FJ. Dietary overlap between jellyfish and forage fish in the northern Gulf of Mexico. Marine Ecology Progress Series. 2018;587:31-40.

2. Baltensperger AP, Huettmann F, Hagelin JC, Welker JM. Quantifying trophic niche spaces of small mammals using stable isotopes (δ15N and δ13C) at two scales across Alaska. Canadian Journal of Zoology. 2015;93(7):579-88.

3. Bastos RF, Corrêa F, Winemiller KO, Garcia AM. Are you what you eat? Effects of trophic discrimination factors on estimates of food assimilation and trophic position with a new estimation method. Ecological indicators. 2017;75:234-41.

4. Baumann C, Bocherens H, Drucker DG, Conard NJ. Fox dietary ecology as a tracer of human impact on Pleistocene ecosystems. PLoS One. 2020;15(7):e0235692.

5. Bernal V, Gonzalez PN, Gordón F, Perez SI. Exploring dietary patterns in the southernmost limit of prehispanic agriculture in America by using Bayesian stable isotope mixing models. Current Anthropology. 2016;57(2):230-9.

6. Berthelsen AS, Søndergaard M, Kiljunen M, Eloranta AP, Lauridsen TL. Pelagic niche shift by fishes following restorations of a eutrophic lake. Hydrobiologia. 2024:1-15.

7. Borray-Escalante NA, Mazzoni D, Ortega-Segalerva A, Arroyo L, Morera-Pujol V, González-Solís J, et al. Diet assessments as a tool to control invasive species: comparison between Monk and Rose-ringed parakeets with stable isotopes. Journal of Urban Ecology. 2020;6(1):juaa005.

8. Bosenbecker C, Bugoni L. Trophic niche similarities of sympatric Turdus thrushes determined by fecal contents, stable isotopes, and bipartite network approaches. Ecology and Evolution. 2020;10(17):9073-84.

9. Brett M, Eisenlord M, Galloway A. Using multiple tracers and directly accounting for trophic modification improves dietary mixing‐model performance. Ecosphere. 2016;7(8):e01440.

10. Brownscombe JW, Shipley ON, Griffin LP, Morley D, Acosta A, Adams AJ, et al. Application of telemetry and stable isotope analyses to inform the resource ecology and management of a marine fish. Journal of Applied Ecology. 2022;59(4):1110-21.

11. Buckner E, Chittaro P, Wood F, Klinger T. Identifying dietary preferences in breeding pigeon guillemot (*Cepphus columba*) using different methods. Northwestern Naturalist. 2022;103(1):42-50.

12. Bury SJ, Peters KJ, Sabadel AJ, Glew KSJ, Trueman C, Wunder M, et al. Southern Ocean humpback whale trophic ecology. I. Combining multiple stable isotope methods elucidates diet, trophic position and foraging areas. Marine Ecology Progress Series. 2024;734:123-55.

13. Calado J, Matos D, Ramos J, Moniz F, Ceia F, Granadeiro J, et al. Seasonal and annual differences in the foraging ecology of two gull species breeding in sympatry and their use of fishery discards. Journal of Avian Biology. 2018;49(1).

14. Catry I, Sampaio A, Silva MC, Moreira F, Franco AM, Catry T. Combining stable isotope analysis and conventional techniques to improve knowledge of the diet of the European Roller *Coracias garrulus*. Ibis. 2019;161(2):272-85.

15. Catry T, Lourenço PM, Lopes RJ, Carneiro C, Alves JA, Costa J, et al. Structure and functioning of intertidal food webs along an avian flyway: a comparative approach using stable isotopes. Functional Ecology. 2016;30(3):468-78.

16. Chiaradia A, Forero MG, McInnes JC, Ramírez F. Searching for the true diet of marine predators: incorporating Bayesian priors into stable isotope mixing models. PloS one. 2014;9(3):e92665.

17. D’Ambra I, Graham WM, Carmichael RH, Hernandez FJ. Fish rely on scyphozoan hosts as a primary food source: evidence from stable isotope analysis. Marine Biology. 2015;162:247-52.

18. Davidson KH, Starzomski BM, El‐Sabaawi R, Hocking MD, Reynolds JD, Wickham SB, et al. Marine subsidy promotes spatial and dietary niche variation in an omnivore, the Keen’s mouse (*Peromyscus keeni*). Ecology and Evolution. 2021;11(24):17700-22.

19. de Moraes CRF, de Attayde JL, Henry-Silva GG. Stable isotopes of C and N as dietary indicators of Nile tilapia (*Oreochromis niloticus*) cultivated in net cages in a tropical reservoir. Aquaculture Reports. 2020;18:100458.

20. Demopoulos AW, McClain-Counts JP, Bourque JR, Prouty NG, Smith BJ, Brooke S, et al. Examination of *Bathymodiolus childressi* nutritional sources, isotopic niches, and food-web linkages at two seeps in the US Atlantic margin using stable isotope analysis and mixing models. Deep Sea Research Part I: Oceanographic Research Papers. 2019;148:53-66.

21. Derbridge JJ, Merkle JA, Bucci ME, Callahan P, Koprowski JL, Polfus JL, et al. Experimentally derived δ13C and δ15N discrimination factors for gray wolves and the impact of prior information in Bayesian mixing models. PloS one. 2015;10(3):e0119940.

22. deVries MS. The role of feeding morphology and competition in governing the diet breadth of sympatric stomatopod crustaceans. Biology Letters. 2017;13(4):20170055.

23. Dromard CR, Bouchon-Navaro Y, Harmelin-Vivien M, Bouchon C. Diversity of trophic niches among herbivorous fishes on a Caribbean reef (Guadeloupe, Lesser Antilles), evidenced by stable isotope and gut content analyses. Journal of Sea Research. 2015;95:124-31.

24. Fernández-Corredor E, Francotte L, Martino I, Fernández-Álvarez FÁ, García-Barcelona S, Macías D, et al. Assessing juvenile swordfish (*Xiphias gladius*) diet as an indicator of marine ecosystem changes in the northwestern Mediterranean Sea. Marine Environmental Research. 2023;192:106190.

25. Flemming SA, van Heezik Y. Stable isotope analysis as a tool to monitor dietary trends in little penguins *E udyptula minor*. Austral Ecology. 2014;39(6):656-67.

26. Gama LR, Fuentes MM, Trevizani TH, Pellizzari F, Lemons GE, Seminoff JA, et al. Trophic ecology of juvenile green turtles in the Southwestern Atlantic Ocean: insights from stable isotope analysis and niche modelling. Marine Ecology Progress Series. 2021;678:139-52.

27. Gaspar C, Giménez J, Andonegi E, Astarloa A, Chouvelon T, Franco J, et al. Trophic ecology of northern gannets *Morus bassanus* highlights the extent of isotopic niche overlap with other apex predators within the Bay of Biscay. Marine Biology. 2022;169(8):105.

28. Gillis AJ, Ceriani SA, Seminoff JA, Fuentes MM. Foraging ecology and diet selection of juvenile green turtles in the Bahamas: insights from stable isotope analysis and prey mapping. Marine Ecology Progress Series. 2018;599:225-38.

29. Giménez J, Marçalo A, Ramírez F, Verborgh P, Gauffier P, Esteban R, et al. Diet of bottlenose dolphins (*Tursiops truncatus*) from the Gulf of Cadiz: Insights from stomach content and stable isotope analyses. PLoS One. 2017;12(9):e0184673.

30. Giraldo C, Ernande B, Cresson P, Kopp D, Cachera M, Travers‐Trolet M, et al. Depth gradient in the resource use of a fish community from a semi‐enclosed sea. Limnology and Oceanography. 2017;62(5):2213-26.

31. Grainger R, Raoult V, Peddemors VM, Machovsky‐Capuska GE, Gaston TF, Raubenheimer D. Integrating isotopic and nutritional niches reveals multiple dimensions of individual diet specialisation in a marine apex predator. Journal of Animal Ecology. 2023;92(2):514-34.

32. Guerrero A, Pinnock A, Negrete J, Rogers T. Complementary use of stable isotopes and fatty acids for quantitative diet estimation of sympatric predators, the Antarctic pack-ice seals. Oecologia. 2021;197:729-42.

33. Gurney KE, Classen HL, Clark RG. Testing for effects of growth rate on isotope trophic discrimination factors and evaluating the performance of Bayesian stable isotope mixing models experimentally: A moment of truth? PloS one. 2024;19(6):e0304495.

34. Hall AG, Avens L, McNeill JB, Wallace B, Goshe LR. Inferring long-term foraging trends of individual juvenile loggerhead sea turtles using stable isotopes. Marine Ecology Progress Series. 2015;537:265-76.

35. Hart JP. Human and dog Bayesian dietary mixing models using bone collagen stable isotope ratios from ancestral Iroquoian sites in southern Ontario. Scientific Reports. 2023;13(1):7177.

36. Hoenig BD, Trevelline BK, Latta SC, Porter BA. Integrating DNA-Based Prey Occurrence Probability into Stable Isotope Mixing Models. Integrative and Comparative Biology. 2022;62(2):211-22.

37. Hopkins III JB, Ferguson JM, Tyers DB, Kurle CM. Selecting the best stable isotope mixing model to estimate grizzly bear diets in the Greater Yellowstone Ecosystem. PLoS One. 2017;12(5):e0174903.

38. Ishikawa NF, Finlay JC, Uno H, Ogawa NO, Ohkouchi N, Tayasu I, et al. Combined use of radiocarbon and stable carbon isotopes for the source mixing model in a stream food web. Limnology and Oceanography. 2020;65(11):2688-96.

39. Jackson AK, Eagles‐Smith CA, Robinson WD. Differential reliance on aquatic prey subsidies influences mercury exposure in riparian arachnids and songbirds. Ecology and Evolution. 2021;11(11):7003-17.

40. Jankowska E, De Troch M, Michel LN, Lepoint G, Włodarska-Kowalczuk M. Modification of benthic food web structure by recovering seagrass meadows, as revealed by trophic markers and mixing models. Ecological Indicators. 2018;90:28-37.

41. Jankowska E, Michel LN, Lepoint G, Włodarska-Kowalczuk M. Stabilizing effects of seagrass meadows on coastal water benthic food webs. Journal of Experimental Marine Biology and Ecology. 2019;510:54-63.

42. Johnson DL, Henderson MT, Anderson DL, Booms TL, Williams CT. Bayesian stable isotope mixing models effectively characterize the diet of an Arctic raptor. Journal of Animal Ecology. 2020;89(12):2972-85.

43. Jones NE, Haxton TJ. Spatial patterns of stable isotopes and trophic ecology in a hydropeaking river. River Research and Applications. 2022;38(5):873-83.

44. Kahma TI, Karlson AM, Liénart C, Mörth C-M, Humborg C, Norkko A, et al. Food-web comparisons between two shallow vegetated habitat types in the Baltic Sea. Marine Environmental Research. 2021;169:105402.

45. Kiszka JJ, Caputo M, Méndez-Fernandez P, Fielding R. Feeding ecology of elusive Caribbean killer whales inferred from Bayesian stable isotope mixing models and whalers’ ecological knowledge. Frontiers in Marine Science. 2021;8:648421.

46. Kundu GK, Kim C, Kim D, Bibi R, Kim H, Kang C-K. Phytoplankton fuel fish food webs in a low-turbidity temperate coastal embayment: A stable isotope approach. Frontiers in Marine Science. 2021;8:751551.

47. Larocque SM, Colborne SF, Fisk AT, Johnson TB. Improving trophic niche and diet resolution of the salmonid community of Lake Ontario using three stable isotopes and multiple tissues. Fisheries Research. 2022;255:106455.

48. Lemoine M, Moens T, Vafeiadou A-M, Bezerra LAV, Lana P. Resource utilization of puffer fish in a subtropical bay as revealed by stable isotope analysis and food web modeling. Marine Ecology Progress Series. 2019;626:161-75.

49. Lewis MC, Sealy JC. Coastal complexity: Ancient human diets inferred from Bayesian stable isotope mixing models and a primate analogue. PLoS One. 2018;13(12):e0209411.

50. Liu A, Mazumder D, Pirozzi I, Sammut J, Booth M. The effect of dietary choline and water temperature on the contribution of raw materials to the muscle tissue of juvenile yellowtail kingfish (*Seriola lalandi*): an investigation using a stable isotope mixing model. Animal Feed Science and Technology. 2021;280:115087.

51. Lloret-Lloret E, Navarro J, Giménez J, López N, Albo-Puigserver M, Pennino MG, et al. The seasonal distribution of a highly commercial fish is related to ontogenetic changes in its feeding strategy. Frontiers in Marine Science. 2020;7:566686.

52. Loizaga de Castro R, Saporiti F, Vales DG, García NA, Cardona L, Crespo EA. What are you eating? A stable isotope insight into the trophic ecology of short-beaked common dolphins in the Southwestern Atlantic Ocean. Mammalian Biology. 2016;81:571-8.

53. Manlick PJ, Petersen SM, Moriarty KM, Pauli JN. Stable isotopes reveal limited Eltonian niche conservatism across carnivore populations. Functional Ecology. 2019;33(2):335-45.

54. Mazoudier SQ, Kingsford MJ, Strickland JK, Pitt KA. Stable isotopes reveal sargassum rafts provide a trophic subsidy to juvenile pelagic fishes. Estuarine, Coastal and Shelf Science. 2023;295:108548.

55. McClain‐Counts JP, Demopoulos AW, Ross SW. Trophic structure of mesopelagic fishes in the Gulf of Mexico revealed by gut content and stable isotope analyses. Marine Ecology. 2017;38(4):e12449.

56. Medina-Contreras D, Arenas-González F, Cantera-Kintz J, Sánchez-González A, Giraldo A. Food web structure and isotopic niche in a fringe macro-tidal mangrove system, Tropical Eastern Pacific. Hydrobiologia. 2020;847:3185-99.

57. Méndez-Salgado E, Chacón-Chaverri D, Fonseca LG, Seminoff JA. Trophic ecology of hawksbill turtles (*Eretmochelys imbricata)* in Golfo Dulce, Costa Rica: integrating esophageal lavage and stable isotope (δ13C, δ15N) analysis. Latin american journal of aquatic research. 2020;48(1):114-30.

58. Merquiol L, Mazzocchi MG, D'Ambra I. The planktonic food web in the Gulf of Naples based on the analysis of carbon and nitrogen stable isotope ratios. Marine Ecology. 2023:e12762.

59. Meyhoff SD, Johnson DL, Ellert BH, Lutes K. Seasonal changes of stable isotope signals in the primary feathers of plains sharp‐tailed grouse. Wildlife Society Bulletin. 2023;47(2):e1412.

60. MICHAHELLIS L. USING A THREE-ISOTOPE BAYESIAN MIXING MODEL TO ASSESS THE CONTRIBUTION OF REFUSE DUMPS IN THE DIET OF YELLOW-LEGGED GULL. Ardeola. 2014;61(2):297-309.

61. Mir-Arguimbau J, Navarro J, Balcells M, Martín P, Sabatés A. Feeding ecology of blue whiting (*Micromesistius poutassou*) in the NW Mediterranean: the important role of Myctophidae. Deep Sea Research Part I: Oceanographic Research Papers. 2020;166:103404.

62. Monteiro S, Ferreira M, Vingada JV, López A, Brownlow A, Méndez-Fernandez P. Application of stable isotopes to assess the feeding ecology of long-finned pilot whale (*Globicephala melas*) in the Northeast Atlantic Ocean. Journal of Experimental Marine Biology and Ecology. 2015;465:56-63.

63. Moscardi BF, Bernal V, Silva Araújo M, Gordón F, Cobos VA, Brachetta‐Aporta N, et al. Diet composition and prey choice in prehistoric human individuals from Northwest Patagonia: An application of species distribution and isotope mixing models. American Journal of Biological Anthropology. 2022;179(4):568-84.

64. Mumby JA, Larocque SM, Johnson TB, Stewart TJ, Fitzsimons JD, Weidel BC, et al. Diet and trophic niche space and overlap of Lake Ontario salmonid species using stable isotopes and stomach contents. Journal of Great Lakes Research. 2018;44(6):1383-92.

65. Nadjafzadeh M, Voigt CC, Krone O. Spatial, seasonal and individual variation in the diet of White‐tailed Eagles Haliaeetus albicilla assessed using stable isotope ratios. Ibis. 2016;158(1):1-15.

66. Nash LN, Kratina P, Recalde FC, Jones JI, Izzo T, Romero GQ. Tropical and temperate differences in the trophic structure and aquatic prey use of riparian predators. Ecology Letters. 2023;26(12):2122-34.

67. Ogilvy C, Constantine R, Bury SJ, Carroll EL. Diet variation in a critically endangered marine predator revealed with stable isotope analysis. Royal Society Open Science. 2022;9(8):220470.

68. Pacioglu O, Zubrod JP, Schulz R, Jones JI, Pârvulescu L. Two is better than one: combining gut content and stable isotope analyses to infer trophic interactions between native and invasive species. Hydrobiologia. 2019;839(1):25-35.

69. Páez‐Rosas D, Rodríguez‐Pérez M, Riofrío‐Lazo M. Competition influence in the segregation of the trophic niche of otariids: a case study using isotopic bayesian mixing models in Galapagos pinnipeds. Rapid Communications in Mass Spectrometry. 2014;28(23):2550-8.

70. Pagani-Núñez E, Renom M, Mateos-Gonzalez F, Cotín J, Senar JC. The diet of great tit nestlings: Comparing observation records and stable isotope analyses. Basic and Applied Ecology. 2017;18:57-66.

71. Pérez-Posada I, Cabanillas-Terán N, Rosas-Luis R, Hernández-Arana HA, Sánchez-Gonzalez A. Isotopic niche shift in the sea urchins *Echinometra lucunter* and *E. viridis* after massive arrivals of Sargassum in the Mexican Caribbean. Regional Studies in Marine Science. 2023;65:103064.

72. Pickett PJ, Dwyer GK, Macqueen A, Holt G, Halliday BT, Barton JL, et al. Using biotracer techniques to uncover consumer diets: A comparison of stable isotopes, fatty acids, and amino acids. Ecosphere. 2024;15(2):e4767.

73. Planas M. Was that my meal? Uncertainty from source sampling period in diet reconstruction based on stable isotopes in a syngnathid fish. Frontiers in Marine Science. 2022;9:982883.

74. Prado P, Baeta M, Mestre E, Solis MA, Sanhauja I, Gairin I, et al. Trophic role and predatory interactions between the blue crab, *Callinectes sapidus*, and native species in open waters of the Ebro Delta. Estuarine, Coastal and Shelf Science. 2024;298:108638.

75. Qin C, Chen P, Sara G, Mo B, Zhang A, Li X. Ecological implications of purple sea urchin (*Heliocidaris crassispina*, Agassiz, 1864) enhancement on the coastal benthic food web: evidence from stable isotope analysis. Marine Environmental Research. 2020;158:104957.

76. Ramirez MD, Avens L, Goshe LR, Snover ML, Cook M, Heppell SS. Regional variation in Kemp’s ridley sea turtle diet composition and its potential relationship with somatic growth. Frontiers in Marine Science. 2020;7:253.

77. Robinson BG, Franke A, Derocher AE. Stable isotope mixing models fail to estimate the diet of an avian predator. The Auk: Ornithological Advances. 2018;135(1):60-70.

78. Rosas-Luis R, Navarro J, Loor-Andrade P, Forero MG. Feeding ecology and trophic relationships of pelagic sharks and billfishes coexisting in the central eastern Pacific Ocean. Marine Ecology Progress Series. 2017;573:191-201.

79. Rossi F, Baeta A, Marques JC. Stable isotopes reveal habitat-related diet shifts in facultative deposit-feeders. Journal of Sea Research. 2015;95:172-9.

80. Saccò M, Blyth AJ, Humphreys WF, Cooper SJ, Austin AD, Hyde J, et al. Refining trophic dynamics through multi‐factor Bayesian mixing models: A case study of subterranean beetles. Ecology and Evolution. 2020;10(16):8815-26.

81. Scholz C, Firozpoor J, Kramer‐Schadt S, Gras P, Schulze C, Kimmig SE, et al. Individual dietary specialization in a generalist predator: A stable isotope analysis of urban and rural red foxes. Ecology and Evolution. 2020;10(16):8855-70.

82. Scriber KE, France CA, Jackson FL. Invasive Apple Snail Diets in Native vs. Non-Native Habitats Defined by SIAR (Stable Isotope Analysis in R). Sustainability. 2022;14(12):7108.

83. Silberberger MJ, Koziorowska-Makuch K, Kuliński K, Kędra M. Stable isotope mixing models are biased by the choice of sample preservation and pre-treatment: Implications for studies of aquatic food webs. Frontiers in Marine Science. 2021;7:621978.

84. Song P-Q, Zhang H-S, Zheng X-Q, Li Y-Y, Lin L-S, Li Y. Trophic plasticity of Bombay Duck *(Harpadon nehereus)* in the South-Central East China Sea based on stable isotope evidence. Frontiers in Marine Science. 2021;8:728773.

85. Sporta Caputi S, Kabala JP, Rossi L, Careddu G, Calizza E, Ventura M, et al. Individual diet variability shapes the architecture of Antarctic benthic food webs. Scientific Reports. 2024;14(1):12333.

86. Swan GJ, Bearhop S, Redpath SM, Silk MJ, Goodwin CE, Inger R, et al. Evaluating Bayesian stable isotope mixing models of wild animal diet and the effects of trophic discrimination factors and informative priors. Methods in Ecology and Evolution. 2020;11(1):139-49.

87. Torres‐Poché Z, Mora MA, Boutton TW, Morrow ME. Diet sources of the endangered Attwater's prairie‐chicken in Texas: evidence from δ13C, δ15N, and Bayesian mixing models. Ecosphere. 2020;11(10):e03269.

88. Tripp-Valdez A, Arreguin-Sanchez F, Zetina-Rejon MJ. The use of stable isotopes and mixing models to determine the feeding habits of soft-bottom fishes in the southern Gulf of California. Cah Biol Mar. 2015;56:13-23.

89. Varela JL, Rojo-Nieto E, Sorell JM, Medina A. Using stable isotope analysis to assess trophic relationships between Atlantic bluefin tuna (*Thunnus thynnus*) and striped dolphin (Stenella coeruleoalba) in the Strait of Gibraltar. Marine environmental research. 2018;139:57-63.

90. Vejříková I, Vejřík L, Čech M, Blabolil P, Peterka J. Variable diet plasticity in Eurasian perch (*Perca fluviatilis*): Current versus seasonal food uptake. Ecology of Freshwater Fish. 2023;32(4):795-803.

91. Velasquez-Vacca A, Seminoff JA, Jones TT, Balazs GH, Cardona L. Isotopic ecology of Hawaiian green sea turtles (*Chelonia mydas*) and reliability of δ13C, δ15N, and δ34S analyses of unprocessed bone samples for dietary studies. Marine Biology. 2023;170(7):81.

92. Wang J, Lu N, Fu B. Inter-comparison of stable isotope mixing models for determining plant water source partitioning. Science of the Total Environment. 2019;666:685-93.

93. Wang P, Zhang F, Liu M, Sun S, Xian H. Isotopic evidence for size-based dietary shifts in the jellyfish *Cyanea nozaki*i in the northern East China Sea. Journal of Plankton Research. 2020;42(6):689-701.

94. Warlick AJ, Ylitalo GM, Neill SM, Hanson MB, Emmons C, Ward EJ. Using Bayesian stable isotope mixing models and generalized additive models to resolve diet changes for fish-eating killer whales *Orcinus orca*. Marine Ecology Progress Series. 2020;649:189-200.

95. Weber AM, Bauer JE, Thomas Watters G. Assessment of nutritional subsidies to freshwater mussels using a multiple natural abundance isotope approach. Freshwater Biology. 2017;62(3):615-29.

96. White AF, Dawson RD. Can diet composition estimates using stable isotope analysis of feathers predict growth and condition in nestling mountain bluebirds (*Sialia currucoides*)? Ecology and Evolution. 2021;11(21):15273-88.

97. Wigginton RD, Van Grootheest C, Spautz H, Grenier JL, Whitcraft CR. Stable isotope mixing models demonstrate the role of an invasive plant in wetland songbird food webs. Applications in Plant Sciences. 2022;10(4):e11486.

98. Wißing C, Rougier H, Crevecoeur I, Germonpré M, Naito YI, Semal P, et al. Isotopic evidence for dietary ecology of late Neandertals in North-Western Europe. Quaternary International. 2016;411:327-45.

99. Wolters J-W, Verdonschot RC, Schoelynck J, Brion N, Verdonschot PF, Meire P. Stable isotope measurements confirm consumption of submerged macrophytes by macroinvertebrate and fish taxa. Aquatic Ecology. 2018;52(4):269-80.

100. Yang Z, Chen X, Zhao N, Tang H, Tao J, Zhang P, et al. The effect of different habitat types and ontogenetic stages on the diet shift of a critically endangered fish species, *Coreius guichenoti* (Sauvage and Dabry de Thiersant, 1874). International Journal of Environmental Research and Public Health. 2018;15(10):2240.

101. Zapata‐Hernández G, Sellanes J, Letourneur Y, Harrod C, Morales NA, Plaza P, et al. Tracing trophic pathways through the marine ecosystem of Rapa Nui (Easter Island). Aquatic Conservation: Marine and Freshwater Ecosystems. 2021;31(2):304-23.

102. Zha J, Wu Y, An Y. Diet analysis of Père David's deer (*Elaphurus davidianus*) based on stable isotope analysis. Wildlife Biology. 2023:e01136.

103. Zhang R, Zhang H, Liu H, Zhao J. Differences in trophic structure and trophic pathways between artificial reef and natural reef ecosystems along the coast of the North yellow Sea, China, based on stable isotope analyses. Ecological Indicators. 2021;125:107476.
