# Supplementary material for: From static to dynamic: Embracing dynamics in isotopic diet estimation
Source: PLoS One. 2025 Aug 26;20(8):e0330327. doi: 10.1371/journal.pone.0330327 (PMC12380277; doi:10.1371/journal.pone.0330327)
Supplement: S2 Appendix — (DOCX) [file pone.0330327.s002.docx]

Appendix 2: Isotopic space effect on maximum bias

The objective of this in-silico experiment was to determine whether the size of the isotopic space affects the magnitude of estimation bias. The isotopic space is defined as the area enclosed by the source polygon, within which the consumer's isotopic values are expected to vary. To test this, an in-silico experiment was conducted using a two-isotope, three-source framework. The dietary scenario assumes that the consumer is at equilibrium, with the diet consisting entirely of source 3. In this experiment, the consumer feeds exclusively on the third source (δ¹³C = 5‰ and δ¹⁵N = 10‰), and the sources are constant over time. The trophic discrimination factor (TDF) is set to 0%, and the elemental concentration is set to 1 for all isotopes and sources. Five different isotopic spaces were tested, each with a single consumer value.

As outlined in the main article, bias was evaluated for each isotopic space across 20 different values of the product λT. The isotopic space was quantified using the Euclidean distance between the two most distant vertices of the source polygon. A larger Euclidean distance indicates a broader isotopic space. In each case, the initial isotopic value of the consumer and its equilibrium signature were placed at the two most distant vertices, ensuring that the Euclidean distance of the isotopic space matched the consumer's distance to equilibrium. The Euclidean distances of the tested isotopic spaces ranged from 2 to 18. These configurations are illustrated in Figure A and detailed in Table 1.

***Table A: Studied isotopic space Maximum Euclidian distance and their associated color***

| **Maximum Euclidian distance** | **Space color** |
| --- | --- |
| 2.0 | Orange |
| 5.0 | Red |
| 8.5 | Blue |
| 11.2 | Pink |
| 18.0 | Green |


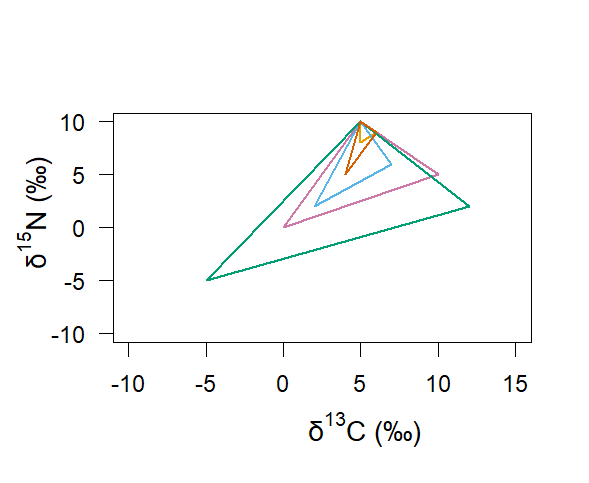


***Figure A: Tested isotopic spaces***

In this setup, the **bias** represents the maximum possible error, as the consumer’s initial isotopic signature was positioned at the point furthest from equilibrium—reflecting a complete (100%) dietary shift. Bias was calculated based on all model solutions that matched the observed consumer signature within a tolerance of **±0.2‰.** The relationship between bias and **λT** is illustrated in the following figure. An interesting point is that all the isotopic **spaces** show very similar bias results when plotted against **λT**, which indicates that the isotopic space has **no impact** on the bias.


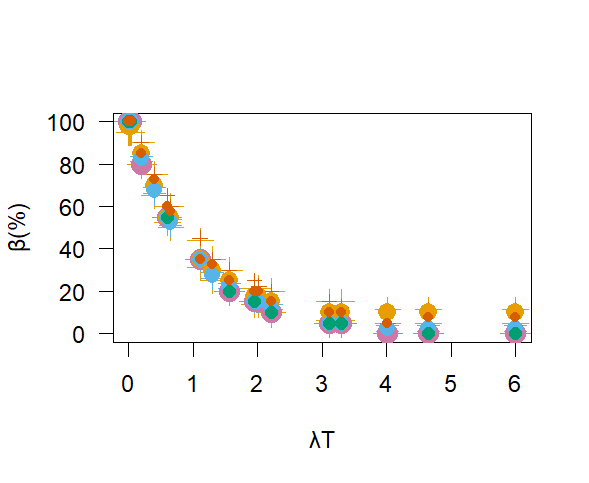


***Figure B: Bias according to λT for the 5 different isotopic spaces. The points represent the median bias and the arrows represents the quartiles. Each color is associated with the represented isotopic space. The points are presented with different sizes to be able to differentiate overlapping points.***
