# Supplementary material for: From static to dynamic: Embracing dynamics in isotopic diet estimation
Source: PLoS One. 2025 Aug 26;20(8):e0330327. doi: 10.1371/journal.pone.0330327 (PMC12380277; doi:10.1371/journal.pone.0330327)
Supplement: S3 Appendix — (DOCX) [file pone.0330327.s003.docx]

Appendix3: Sources dynamics in in-silico experiments

To assess the effect of varying source values on mixing model estimates, five different scenarios of source variation were modeled. In the **first scenario (Figure 1),** the sources were kept constant over time. The **second scenario** involved two dynamic sources (sources 2 and 3) for Carbon, while the rest remained stable, with their values similar to those in Scenario 1. The dynamic sources became closer over time, shrinking the source polygon (Figure 2). In the **third scenario**, sources 2 and 3 were again dynamic for the first isotope, but their values decreased over time (Figure 3). The **fourth scenario** mirrored Scenario 3 in terms of dynamic sources but saw the values of sources 2 and 3 increase over time (Figure 4). Finally, the **fifth scenario** was similar to Scenario 4, but with a decrease in the dynamic sources' values for the first isotope over time (Figure 5).


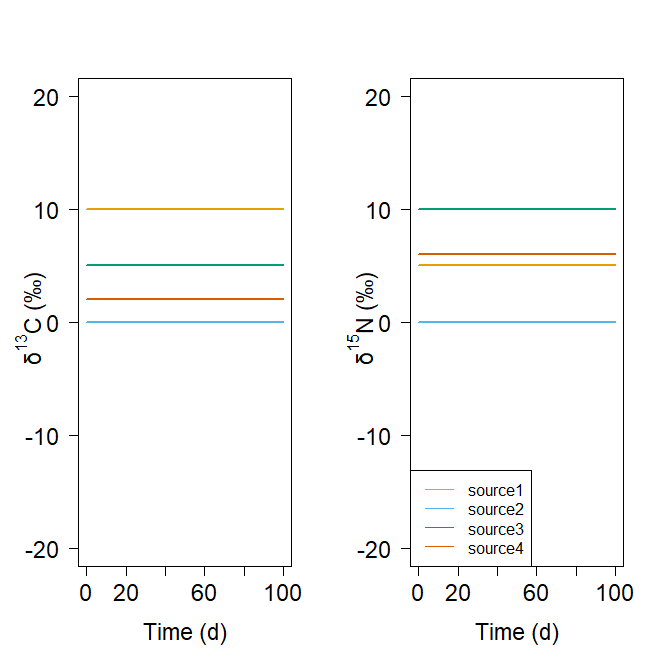


*Figure A:* Source isotopic values across time in Scenario 1 (100-day period).


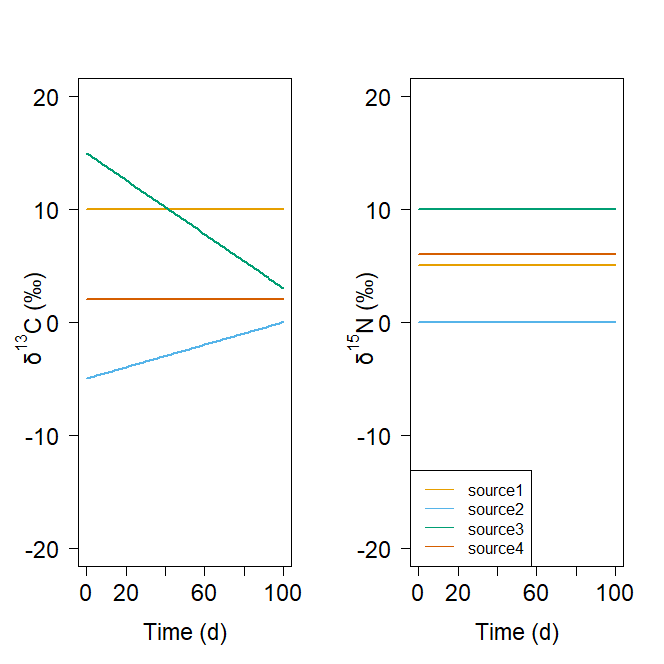


*Figure B: Source value over time in scenario 2 for each isotope and a period of 100 days.*


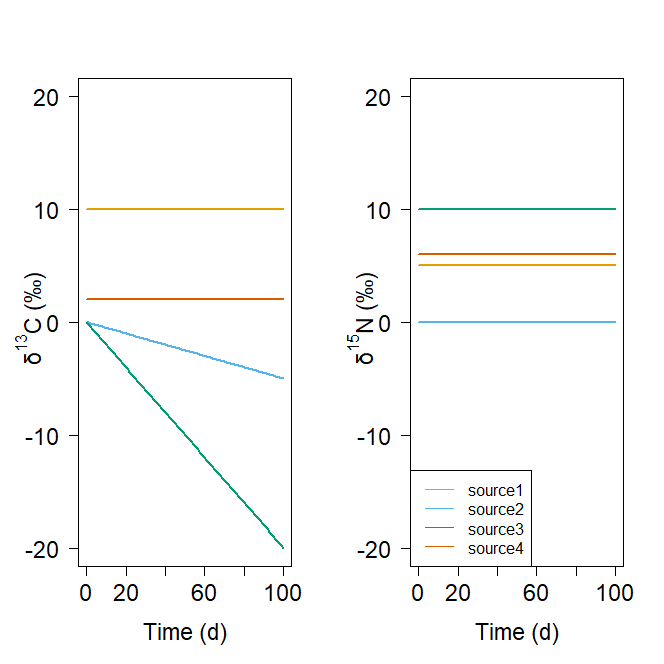


*Figure C: Source value over time in scenario 3 for each isotope and a period of 100 days.*


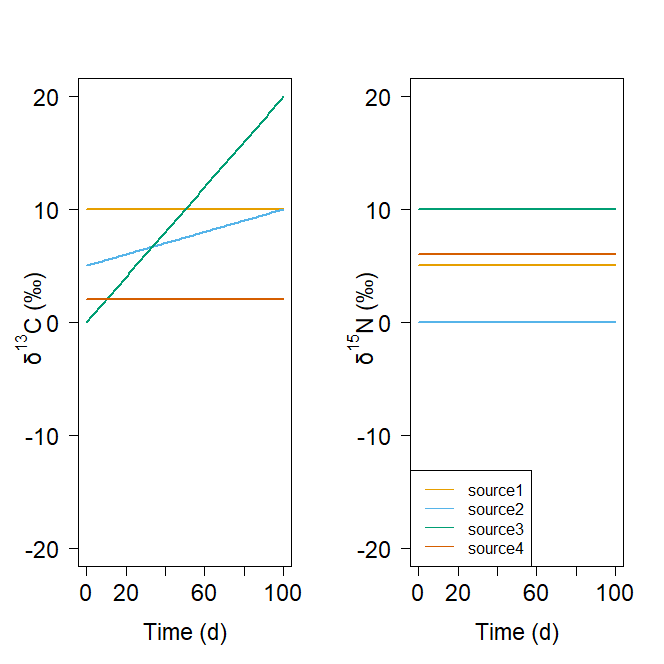


*Figure D: Source value over time in scenario 4 for each isotope and a period of 100 days.*


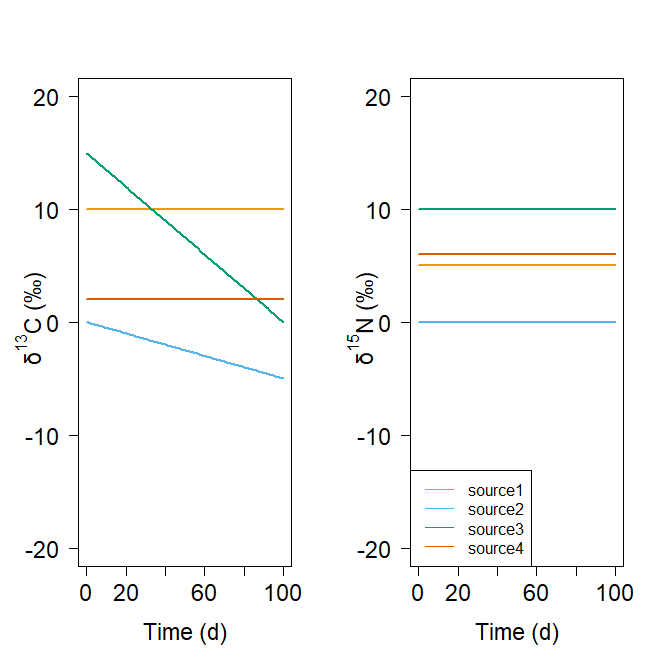


*Figure E: Source value over time in scenario 5 for each isotope and a period of 100 days*
