# Supplementary material for: From static to dynamic: Embracing dynamics in isotopic diet estimation
Source: PLoS One. 2025 Aug 26;20(8):e0330327. doi: 10.1371/journal.pone.0330327 (PMC12380277; doi:10.1371/journal.pone.0330327)
Supplement: S4 Appendix — (DOCX) [file pone.0330327.s004.docx]

Appendix4: Applied dynamic mixing model diet contributions over time

This section presents the contributions of each source to the diet over time in the applied Dynamic Mixing Model (DMM) in silico experiment. The simulated dataset spans 500 days. Throughout the experiment, **Source 1** does not contribute to the diet at any point, maintaining a 0% contribution. **Source 2** oscillates between 100% and 0%, following a sinusoidal function as described below.

$\begin{aligned} s2_{contrib}\left( t \right)=0.5*sin\left( 0.025*t \right)+0.5\#\left( 1 \right) \end{aligned}$urce 3 follows a sinus function as well that is describe hereafter:

$$\begin{aligned} s3_{contrib}\left( t \right)=1-s2_{contrib}\left( t \right)\#\left( 2 \right) \end{aligned}$$

The evolution of each contribution over time is detailed in Figure A.


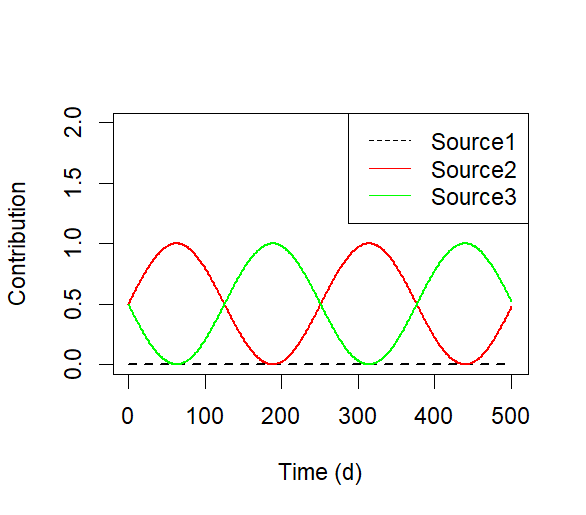


*Figure A: Source contribution over time*
