# Supplementary material for: From static to dynamic: Embracing dynamics in isotopic diet estimation
Source: PLoS One. 2025 Aug 26;20(8):e0330327. doi: 10.1371/journal.pone.0330327 (PMC12380277; doi:10.1371/journal.pone.0330327)
Supplement: S5 Appendix — (DOCX) [file pone.0330327.s005.docx]

## Appendix5: dataset Inger et al.,2006 and mixing model outputs

## Blood-cell tissue

This section presents the dataset used for the case study in the article (Table A & B). The data is sourced from (Inger et al., 2006) and was retrieved from the MixSIAR example database *(Stock et al., 2018).* It represents the isotopic signature of blood-cell tissue. A visual representation of the dataset is provided, with source values corrected by trophic discrimination factor (TDF) values (Figure A).

*TableA: Isotopic Signatures of the blood-cell tissue for geese*

| Consumer data (blood cell) | | | |
| --- | --- | --- | --- |
| Sampling date | Time (d) | δ^13^C (‰) | δ^15^N (‰) |
| October 2003 | 0 | -12.64 | 9.09 |
| January 2004 | 92 | -14.63 | 10.30 |
| February 2004 | 123 | -20.07 | 10.51 |
| November 2004 | 396 | -13.99 | 9.55 |
| December 2004 | 426 | -12.63 | 10.30 |
| January 2005 | 457 | -21.28 | 9.17 |
| February 2005 | 488 | -26.33 | 8.84 |
| April 2005 | 547 | -27.56 | 8.09 |
| October 2005 | 730 | -12.68 | 8.49 |

*Table B: Isotopic signatures, concentrations and TDF of geese possible food sources*

| Sources data | | | | | | |
| --- | --- | --- | --- | --- | --- | --- |
| Source | δ^13^C (‰) | δ^15^N (‰) | Carbon concentration | Nitrogen concentration | TDF (‰) Carbon | TDF (‰) Nitrogen |
| Zoostera | -11.7 | 6.49 | 0.36 | 0.03 | 1.63 | 3.54 |
| Grass | -30.88 | 4.43 | 0.4 | 0.35 | 1.63 | 3.54 |
| Ulva | -11.17 | 11.19 | 0.21 | 0.02 | 1.63 | 3.54 |
| Enteromorpha | -14.06 | 9.82 | 0.18 | 0.01 | 1.63 | 3.54 |


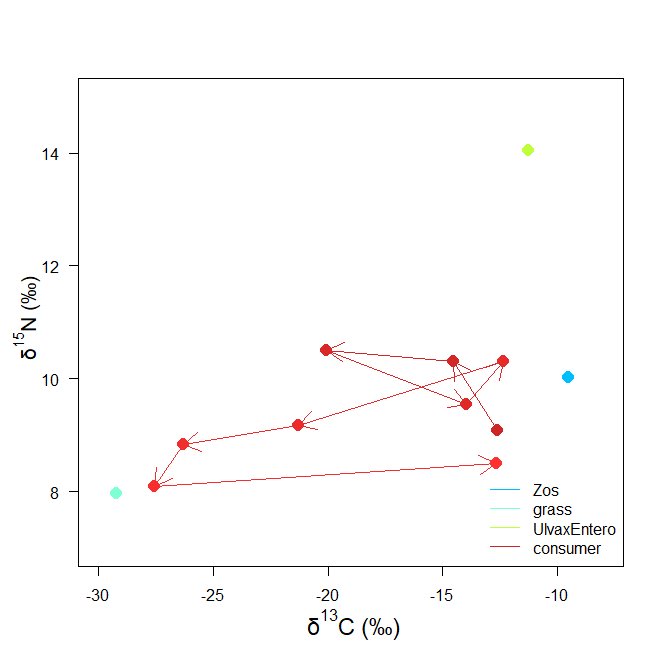


**Figure A: Biplot of TDF-corrected source signatures (green/blue) vs. consumer blood cell signature over time (red), with arrows depicting the chronological evolution of the consumer.**

## Plasma tissue

The plasma dataset was extracted from the Inger at al.,2006 article using repeated measures with the PlotReader application (Table C). A visual representation of the dataset is proposed with the source values corrected by TDF values (Figure B).

*TableC: Isotopic Signatures of the plasma tissue for geese*

| Consumer data (plasma) | | | |
| --- | --- | --- | --- |
| Sampling date | Time (d) | δ^13^C (‰) | δ^15^N (‰) |
| October 2003 | 0 | -12.09 | 10.32 |
| January 2004 | 92 | -15.79 | 11.77 |
| February 2004 | 123 | -21.00 | 10.60 |
| November 2004 | 396 | -12.42 | 11.00 |
| December 2004 | 426 | -14.84 | 12.12 |
| January 2005 | 457 | -20.23 | 10.82 |
| February 2005 | 488 | -25.70 | 8.97 |
| April 2005 | 547 | -27.32 | 8.30 |
| October 2005 | 730 | -10.80 | 9.41 |


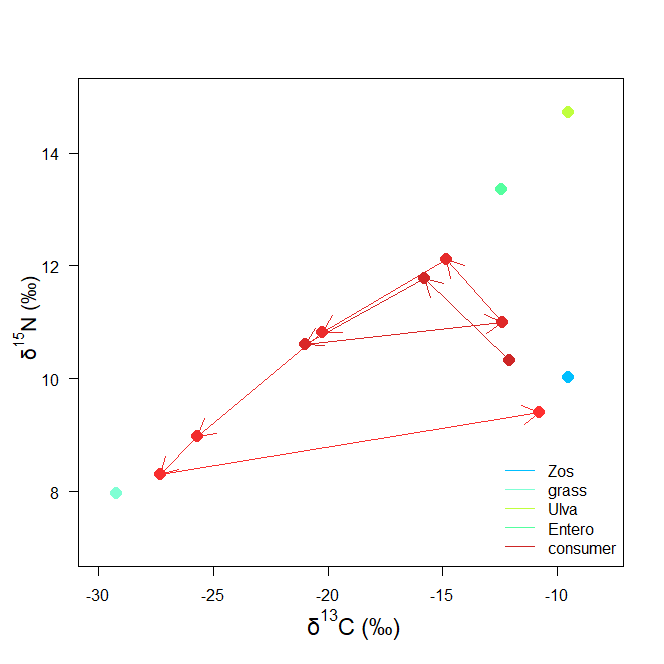


**Figure B: Biplot of TDF-corrected source signatures (green/blue) vs. consumer blood cell signature over time (red), with arrows depicting the chronological evolution of the consumer.**

## Contribution of dietary sources over time: solution of the SMM/DMM (50 best solutions) for the plasma tissue


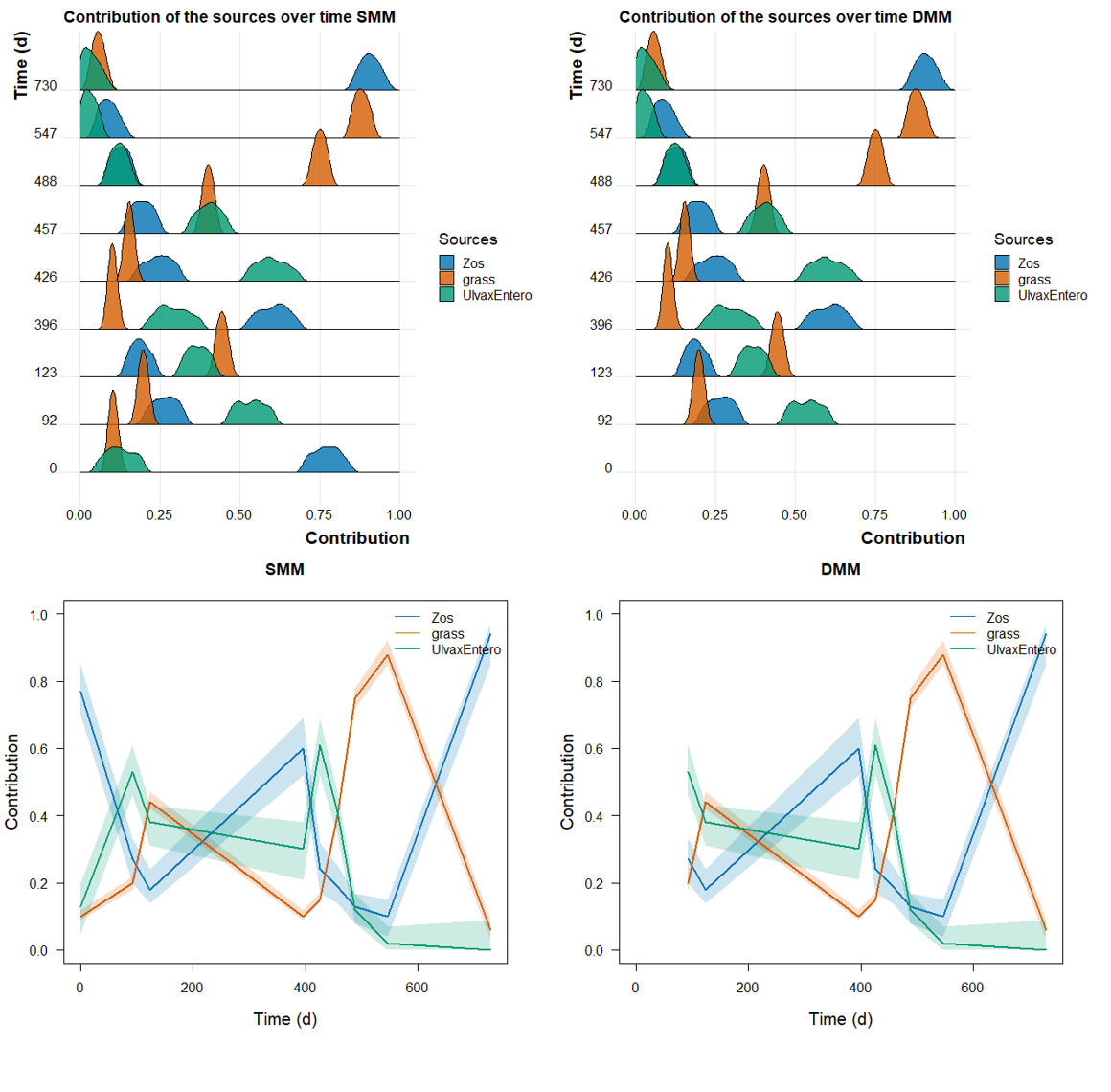


**Figure C: Density plots and evolution over time of sources contribution estimates by SMM and DMM for the plasma tissue**

The figure shows very similar estimates for the 2 models that can be explained by a very high λ value (0.3d^-1^) resulting of a very low bias for the SMM.

## References

Inger, R., Ruxton, G. D., Newton, J., Colhoun, K., Robinson, J. A., Jackson, A. L., & Bearhop, S. (2006). Temporal and intrapopulation variation in prey choice of wintering geese determined by stable isotope analysis. *Journal of Animal Ecology*, 1190-1200.

Stock, B. C., Jackson, A. L., Ward, E. J., Parnell, A. C., Phillips, D. L., & Semmens, B. X. (2018). Analyzing mixing systems using a new generation of Bayesian tracer mixing models. *PeerJ*, *6*, e5096.
