# Supplementary material for: From static to dynamic: Embracing dynamics in isotopic diet estimation
Source: PLoS One. 2025 Aug 26;20(8):e0330327. doi: 10.1371/journal.pone.0330327 (PMC12380277; doi:10.1371/journal.pone.0330327)
Supplement: S6 Appendix — (DOCX) [file pone.0330327.s006.docx]

Appendix 6: Additional results from the in-silico experiments

Results for the source effect for diets 2,3 and 4 tested in-silico. The second diet is characterized as a constrained system (2 isotopes 3 food sources) and the real diet is composed of 15% of source 1, 45% of source 2 and 40% of source 3. The same effect can be observed here than the first diet. There is a source effect as the bias is higher when the sources vary over time than when they are constant. Also, there is a reduction of this additional bias for the SMM_delta. (Fig A).


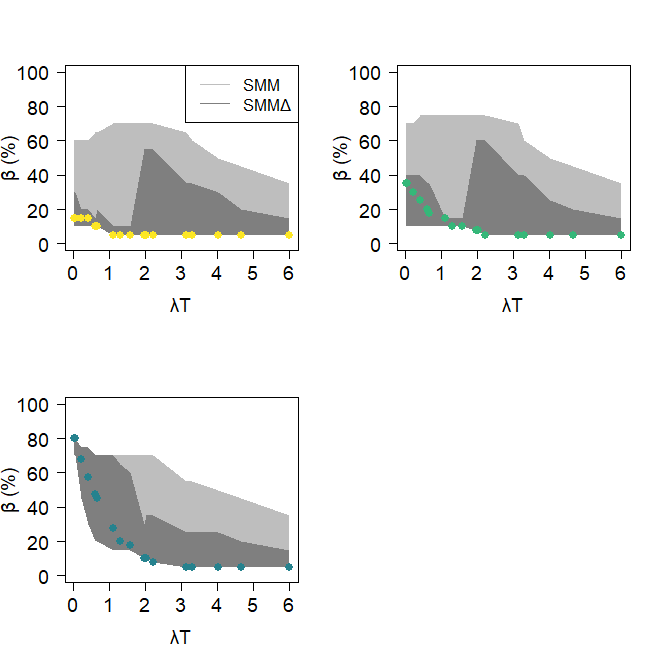


*Fig A: source effect for the second diet. The colored dots are the bias for the four selected initial values when the sources are constant over time and the grey areas represent the bias for the same initial values when the sources are dynamic.*

The third diet is characterized as an un-constrained system (isotopes for 4 food sources), and the real diet consists of 20% of source 1, 20% of source 2, 40% of source 3 and 20% of source 4. In figure B, the system is unconstrained and the source effect is harder to observe. However, there is still possible to observe a light reduction of the additional bias caused by the source dynamics in the SMM_delta (Fig B).


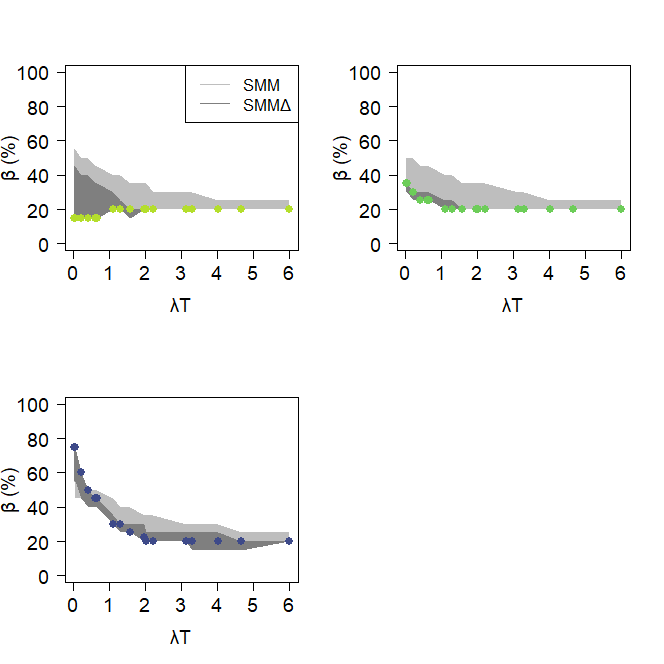


*Fig B: source effect for the third diet. The colored dots are the bias for the four selected initial values when the sources are constant over time and the grey areas represent the bias for the same initial values when the sources are dynamic.*

The fourth diet is characterized as an un-constrained system (isotopes for 4 food sources), and the real diet consists of 100% of source 3. In figure C, the system is unconstrained and the source effect is harder to observe. However, there is still possible to observe a light reduction of the additional bias caused by the source dynamics in the SMM_delta (Fig C).


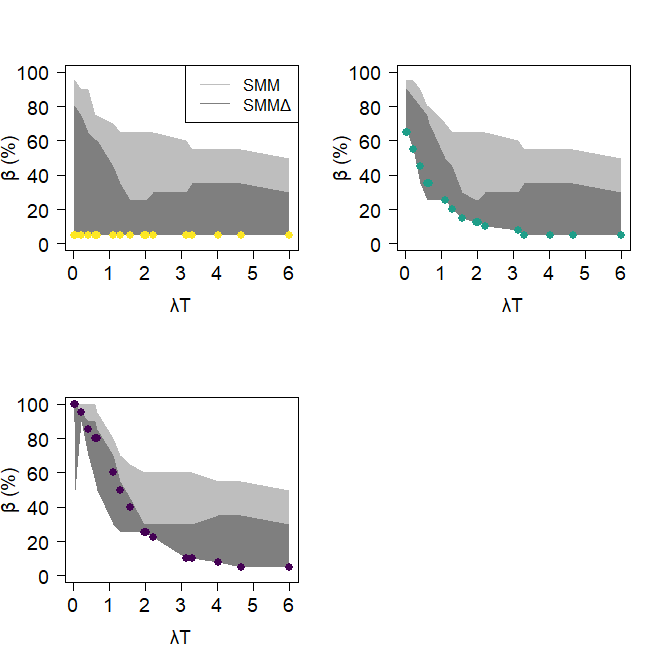


*Fig C: source effect for the fourth diet. The colored dots are the bias for the four selected initial values when the sources are constant over time and the grey areas represent the bias for the same initial values when the sources are dynamic.*
