# Supplementary material for: From static to dynamic: Embracing dynamics in isotopic diet estimation
Source: PLoS One. 2025 Aug 26;20(8):e0330327. doi: 10.1371/journal.pone.0330327 (PMC12380277; doi:10.1371/journal.pone.0330327)
Supplement: S7 Appendix — (DOCX) [file pone.0330327.s007.docx]

# Appendix 7: Comparison of the Dynamic Mixing Model (DMM) and MixSIAR

| Feature | Dynamic Mixing Model (DMM) | MixSIAR (Bayesian Static Mixing Model) |
| --- | --- | --- |
| Framework | Frequentist, deterministic | Bayesian, probabilistic |
| Time dynamics | ✅ Yes (Ordinary Differential Equation-based model over time `t`) | ❌ No (Isotopic equilibrium only, static diet assumed) |
| Source correlation | ❌ Not modeled (sources treated as determined points) | ✅ Yes (sources modeled with multivariate normal distribution capturing δ¹³C–δ¹⁵N correlations) |
| Model Uncertainty quantification | ⚠️ Partial: uncertainty estimated on the basis of combinations compatible with the data (mean + quartiles), but without propagation of statistical error. | ✅ Yes: fully integrated uncertainty via MCMC |
| Data based Error structure | ❌ Not included (no propagation of uncertainty from sources, TDFs, or consumer data) | ✅ Yes (uncertainty is propagated into dietary estimates via MCMC taking account of sources, TDF and consumer measurements) |
| Equilibrium assumption | Optional | Required |
| Estimation output | Set of best-fitting source contributions | Full posterior distributions of source contributions (with credible intervals) |
| Multiple isotopes | ✅ Yes | ✅ Yes |
| Source concentration weighting | ✅ Yes (`q_i`) | ✅ Yes (via concentration dependence option) |
| Time-varying diet | ✅ Yes (via p₁(t), δX₁(t), etc.) | ❌ No |
| Designed for time series | ✅ Yes (diet estimated between sampling dates) | ❌ No (single time point estimation only) |
| Numerical solution | ✅ Yes (using `deSolve` package for Ordinary Differential Equations) | Not applicable |
| Replication of results | ✅ Deterministic, reproducible via script | ✅ Probabilistic, but reproducible with fixed seeds |
| Software implementation | R script (https://github.com/Emilie-Cath/DMM) | R package `MixSIAR` |
| Model validation | Estimation error metric only | Posterior predictive checks, diagnostics (Gelman-Rubin, Geweke, trace plots) |
| Customizability | High, with flexibility to input time-varying data | Moderate, limited to model structures in MixSIAR |
