# Supplementary material for: From static to dynamic: Embracing dynamics in isotopic diet estimation
Source: PLoS One. 2025 Aug 26;20(8):e0330327. doi: 10.1371/journal.pone.0330327 (PMC12380277; doi:10.1371/journal.pone.0330327)
Supplement: Attachment — Submitted filename: Review PONE-D-24-58834.pdf [file pone.0330327.s008.pdf]

Review PONE-D-24-58834

“From Static to Dynamic: Embracing dynamics in Isotopic Diet Estimation”

General remarks

The manuscript aims to describe and analyze bias in mixing models caused by temporal dynamics and to develop a dynamic mixing model. The authors also discuss the influence of the isotopic turnover rate and sampling frequency on the accuracy of mixing models both under the steady-state hypothesis and in the dynamical approach. The manuscript's focal point is the discussion of biases arising due to the absence of temporal dynamics in said models. This is an interesting topic belonging to a broader range of problems related to the estimation of biases and uncertainties in the modelling of stable isotope mixing, rarely discussed in the literature in sufficient detail.

As the SMM calculations typically involve an underdetermined system, i.e. the number of potential food sources typically exceeds the number of measured isotopic signatures, the careful consideration of correlations and uncertainties is crucial and remains a challenging topic both conceptually and in terms of computational complexity. The resulting probability distributions of fractions describing contributions to the mixture from various sources are usually broad and highly correlated, thus preventing drawing any conclusions from central values (mean or median) alone. Additionally, particularly in the studies of trophic networks, there are numerous processes influencing the isotopic composition of tissues (e.g.: trophic discrimination factor, isotopic turnover rate or element concentrations), which results in a high number of model parameters, further increasing the calculation complexity of computation and error estimation.

Following the above consideration, in my view, the manuscript contains several serious flaws that undermine its conclusions, making it unsuitable for publication in a journal. My concerns with the presented methodology primarily relate to the bias calculation method and, more importantly, the implementation of mixing models, which fails to account for correlations. This omission results in an incomplete and potentially misleading discussion of systematic uncertainties.

Regarding the manuscript’s readability, it requires significant improvement. The structure feels chaotic and the text is often unclear, with issues in grammar and typos that disrupt readability. To enhance clarity and coherence, the authors should refine the language, avoid technical jargon, improve the logical flow, and ensure proper adherence to typographical standards. A thorough revision is needed to achieve acceptable presentation standards.

Based on all these factors, it is impossible for me to approve this manuscript. I should however state that the topic of biases in mixing models is very much a worthy pursuit and I encourage the authors to not abandon the topic and try to repeat the analyses with refined methodology.

Below, I outline key criticisms and suggestions for improvement in the hope that they will not discourage the authors but rather contribute to a deeper understanding of the subject.

Criticism

Introductory section

The article makes a case that the majority of studies involving SIMMs published in 2014-2024 do not address the potential temporal changes in analyzed diets. The introductory section leaves the reader with the impression that these studies are faulty and the wrong assumption is made about the steady state of the system. This is a major criticism towards 75% of 103 published papers and in my opinion, this criticism is too strong and not motivated well enough in the manuscript. The steady-state assumption may be an accurate approximation for a given system and thus the temporal dependence is not simply overlooked. In the current formulation, the manuscript appears to undermine the competence of many authors (which is not a problem in itself, but then it requires much more information).

Methodology - calculation of bias

I do not find the method of bias calculation presented in the manuscript sufficiently accurate.
The definition of the β_S factors in Eq. 7 that excludes a single source S is justified with the remark that “calculating bias for every source in the system simultaneously [...] the biases would sum to zero“, which I cannot agree with. While the statement is technically true, I see no reason for selecting such a definition instead of $\beta_i = p_i - \hat{p_i}$. Using this definition, the number of β parameters remains the same, while at the same time, we obtain information on the directionality of the bias for each source individually. Alternatively, if an aggregate bias for the whole system is of interest, one could sum the absolute values or the squares of the $p_i - \hat{p_i}$, this way avoiding the trivial zero-sum. The definition of the β_S factors used in the manuscript is confusing and difficult to interpret.
In my opinion, the calculation of bias must account for the width of the probability distribution of the solutions to the mixing problem. The significance of the bias can only be inferred when compared to the width of such distribution.

Methodology - implementation of mixing models

The implementation of the mixing models uses a so-called “brute force” as finding the solution requires generating all potential combinations of source contributions (with user-defined precision). The computational complexity thus scales with the power of the dimensionality of the problem, which for the typical real-world use cases may rapidly explode. However, a significantly greater concern comes from the fact that the correlations are disregarded completely and these

are absolutely crucial in any discussions involving uncertainties of the mixing models. As already stated, the probability distributions of fractions describing the solutions to the mixture from various sources are usually broad, skewed and highly correlated. The central values alone cannot provide sufficient information to draw conclusions regarding any type of uncertainty (both statistical and systematic). Additionally, as mentioned in the previous paragraph the bias should be studied in relation to the statistical uncertainty, which is completely absent in presented studies.

The community standard in the analyses of isotope mixing is utilizing the Bayesian models that ensure more efficient convergence and can reliably describe the correlation between mixing fractions (authors discuss this in the last paragraph of the article). Moreover, there is a type of existing Bayesian models that would serve as an excellent starting point for the kind of analysis that was attempted in this study - the Markov Chain Monte Carlo algorithm with covariates. Currently, there exists an implementation in R - MixSIAR (Stock et al. PeerJ, 6:e5096, 2018. doi: 10.7717/peerj.5096). A similar analysis may also be carried out using the FRAME model implemented in Python (Lewicki et al., PLOS ONE, 17(11), e0277204), where the user may define a custom mixing model equation with an arbitrary number of parameters.

Bias depending on diet switch

(lines 376-388)

The effects of the diet switch discussed in this section depend trivially on the initial conditions of designed in-silico experiments.

“maximum bias caused by the switch effect is influenced by the Euclidean distance between the consumer's signature prior to the diet switch and its equilibrium state“

This is trivial, similarly to the different behaviour for diets 1, 2 and 3 as explained below.

“In the third diet scenario (Fig. 5B), the system is unconstrained, involving four food sources for two isotopes. Here, no clear relationship between the distance to equilibrium and the switch effect is observed.“

That’s because the spread of the distance to equilibrium is much narrower! The distances reach up to about 11 in diet 1, 8 in diet 2 and close to 7 (guessing from the plot) in diet 3. This is a feature of the initial conditions and the lack of a clear relationship is its trivial consequence. The conclusion is misleading - as if depending on a diet a different behaviour of diet switch effect is to be observed, which is definitely not true.

“Unlike the constrained scenarios (Fig. 4, Fig. 5A), there is no consistent reduction in bias with λT. In this case, the bias remains elevated until the λT value reaches 5, after which it rapidly declines to 0 when $\lambda T > 5$.”

This statement, while not wrong, is also misleading. The observed dependence on λT is once again a trivial consequence of initial conditions. What we have in reality, is the 2-dimensional phase space in λT and Euclidean distance and the ‘bias’ is the result of model calculation for a

given pair of λT and distance. The quoted statement, as well as the previous one, is a consequence of the uneven coverage of the λT-distance phase space across the 3 different diets and nothing more. In fact, in terms of visualization of the results, a much clearer message could be conveyed if λT and distance were denoted on the axes and the bias (as a result) shown with a colour scale or similar. This way, also the uneven coverage of λT-distance phase space would become visible immediately.

In-silico experiment with sinusoidal dependence on time of consumer signatures

(lines 302-321)
The preparation of the experiment raises a number of questions requiring further clarification. Why even consider 3 sources if one of the contributions is set at zero? This creates an unnecessary confusion.
Further, it is not clear how (and why) Eq. 5 was used to generate the consumer isotopic signatures. Was it solved analytically in the form of $d\delta X/dt = \lambda(\delta X_{eq}(t) - \delta X)$, where $\delta X_{eq}(t)=\sum q_i(t)\delta X_{\mathrm{i}}(t)$ and λ=const? If a numerical solver was used, then what was the number of free parameters? As I understand, both $q_i(t)$ and $\delta X_{\mathrm{i}}(t)$ are given, so the $\delta X(t)$ can be calculated directly from Eq.3 - why then not use it?
Moreover, it is not clear how Eq. 5 was used to obtain a solution for the randomly sampled dates. Line 327 reads that “the same model parameters as the environmental setup” were used - does it mean that the $\delta X_{\mathrm{i}}(t)$ and λ=const were treated as known parameters and the $q_i(t)$ was obtained using the numerical solver? Then again, my question is why Eq. 3 was not used instead?

325-326: *“with linear interpolation applied between two sampled points to approximate the source dynamic”*
This approximation is not justified for larger time intervals. Perhaps, it was the authors’ intention to show that there is no gain from using DMM in such cases, but then the discussion in lines 415-425 does not reflect that. Any considerations for λT>2 should be trivially dismissed - the linear approximation of time dependence renders the DMM results useless for longer time intervals between the samples.

Geese case study

355-358
There is no illustration of the available data in the $\delta^{13}C$ vs $\delta^{15}N$. The results in Fig. 9 are difficult to interpret without this kind of plot.
“λT value (which is the time in days between two samplings multiplied by the turnover rate)”
This is a mistake - the T is the time elapsed from the diet switch. The sampling frequency is uncorrelated with the time of the diet switch.
“We hypothesized that the DMM results closely approximated the geese’s real

diet and used these results as a reference to evaluate potential biases in the SMM estimates."
The obtained dependence of bias vs λT (Fig. 9) is a direct consequence of the wrong assumption discussed above. It was demonstrated earlier in the manuscript that the differences between DMM and SMM vanish for high λT so that part of the plot is not surprising. However, at small λT, the results are biased towards $\delta X(0)$ due to overestimated $e^{-\lambda t}$ factor (Eq. 3).
Considering the above, I cannot agree with the presented observations.

Discussion and Conclusions

I refrain from commenting on the discussion and conclusions in the final sections of the manuscript. The authors aim to demonstrate the shortcomings of static models compared to the dynamic ones, however, the reasoning is based on flawed methodology and thus a meaningful discussion can only be held once the fundamental analytical flaws and weaknesses in the design are ruled out first.

Other remarks

- 41: "The analysis of stable isotope is a widely used technique in ecology"
  Too wide of a statement - did you mean the modelling of stable isotope mixing? Or more generally, the studies of isotopic composition of tissues?
- 41-42: Both sentences would benefit from adding citations of relevant review literature.
- 60-63: While the isotopic turnover rate is indeed missing in mixing models, it is not "overlooked", but rather the authors assume that the incorporation time window is short enough in the context of their studies. Please remove the statement or reformulate it.
- 63-64: "Other aspects of isotopic systems are also bound to time dynamics, such as sources and consumer signatures"
  This statement is not precise enough. Please name explicitly what other processes may affect the isotopic signatures of sources and consumer tissues.
- 64-65: "observed and demonstrated"
  It is not clear from the context what is the difference between an observation and the demonstration.
- 63-67: The discussion of potential errors due to the steady state hypothesis is very important in the context of this article. I would be interested in a more elaborate description of observed biases in [8,15-18] (magnitude, statistical significance, influence on the conclusions).
- 69-71: "accounting for source variability by measuring the sources' signatures over a time-window relative to the isotopic incorporation before measuring the consumer signatures"
  I cannot understand this sentence. And in consequence, I also do not understand what is meant by "this disparity" in the next sentence.

Editorial remarks

(only for the introductory section)

- 16: “based on the hypothesis of **stable** isotopic systems **remains stable** over time”. -> verb problem (remaining), repetition of “stable”
- 17: “referred” -> “referred to”
- 18: “ —” remove space
- 23-24: “this study **aims to** (...) **developing** a dynamic mixing model capable of accounting for isotopic dynamics” -> verb problem
- 39: “A time window” -> article “a” not needed
  “Concentration in element on source” -> “in” and “on” seem to be misused.
  “Contribution of source i to diet” -> I would explicitly write “fraction” to avoid ambiguity
- 44: “(referred also to Table 1 for acronyms and notations)” -> grammar problems
- 46: “difference **with** the mixing” -> with respect to
- 50: The phrase "when both equilibrate" is somewhat ambiguous. It could be clarified to specify that it refers to the isotopic signature in the consumer's tissues reaching a stable state after consuming the diet over time.
- 52: “time lag” sounds too informal.
- 54-55: “λ (...) can be also expressed as a function of the half-life” -> then, the inline equation should rather be: $\lambda = \log(2)/t_{1/2}$.
- 56: “using a temporal setting” -> The statement is too vague.
- 59: “dynamic” -> dynamics?
- 61: “λ seems somehow overlooked in diet studies“ -> too informal
- 66 and 68: repeated phrase “these errors”
