# Supplementary material for: From static to dynamic: Embracing dynamics in isotopic diet estimation
Source: PLoS One. 2025 Aug 26;20(8):e0330327. doi: 10.1371/journal.pone.0330327 (PMC12380277; doi:10.1371/journal.pone.0330327)
Supplement: Attachment — Submitted filename: Response to Reviewers.docx [file pone.0330327.s009.docx]

**From Static to Dynamic: Embracing Dynamics in Isotopic Diet Estimation**

Emilie Cathelin^1*^, Sebastien Lefebvre^1^, Carolina Giraldo^2^

This is the answers to the reviewers and Editor ‘s comments. Hereafter in the document, reviewers and editor’s comments will appear in black while the answers will appear in blue. Text appearing crossed was removed from the manuscript and text appearing underlined was added to the manuscript. This document presents the response to reviews of reviewer 1 followed by the answers from reviewer 2 and finally the reviews from the Editor.

To facilitate the review of this document, in the response to the Editor, any indication of lines referring to the present document is indicated in orange.

Reviewer #1: This study sets out to quantify and analyze the bias associated with different stable isotope mixing model approaches, and compare the bias of standard static mixing models with models incorporating information about turnover dynamics, and a dynamic model incorporating temporal isotopic dynamics that was developed within the study. I believe the questions adressed by the paper are highly relevant and fill an important knowledge gap in isotope ecology by implicitly adressing the question of temporal dynamics, in particular variation in the lamda parameter.
The paper is overall well written, but could profit from a more streamlined discussion and a better incorporation of relevant literature.

We thank the reviewer for this feedback. Regarding the discussion, we addressed specific comments made (details below). The discussion was then reorganized to eliminate repetitions and enhance clarity. The previous version of the discussion and conclusion were divided into four parts: (1) Comparison between DMM and SMMs, (2) About the importance of considering the isotopic turnover and sampling/experiment time (λT) in isotopic studies, (3) Field application with review comparison and (4) Conclusion: Limits and further perspectives.

These have now been re-organized as follows:

- We removed the limitations from the conclusion and created a new section in the discussion titled “DMM limitations” (line 663) where we included points previously mentioned in the comparison DMM/SMMs

Some paragraphs from the comparison SMM/DMM were moved to the Field application part.

- Line 604: Field application with review comparison was renamed “Mixing models and bias in the field”
- the paragraph assessing efficiency of Philips recommendation was moved line 636-645
- The paragraph about the applied DMM and contextualization with results was moved lines 614-635
- The paragraph confronting bias and mixing model was a repetition and was removed.

We then changed transitions sentences to re-articulate the paragraphs between themselves.

- Line 506-513 were moved line 518 to 531. To link the two paragraphs together we added line 524-525“. The *in-silico* environment used in this analysis demonstrates the robustness of the DMM.”
- Paragraph 536-543 was linked to the previous one (line 532-536)
- To match the paragraphs about the difficulty of temporal sampling and applicability of DMM to such dataset, we removed the following sentence lines 614-615: “The results from the applied DMM demonstrate its effectiveness under field sampling conditions, showcasing the type of inputs commonly observed in applied diet stable isotope studies.” And changed it to line 614: “The applicability to the DMM depends on the type of dataset.”
- To link the paragraph about Phillips recommendation efficiency and how much it is respected in literature we changed the sentence line 646: “Recommendations made by Phillips et al. [21] aim to minimize bias in isotopic field applications.” By “These recommendations aim to minimize…”

See several comments from both reviewers 1 and 2 for the addition and incorporation of literature. The number of cited articles in the previous version was 61 and is now 83.

The introduction is well written, and conveys the knowledge gaps and the relevance of the questions posed in the paper. However, it might be worthwhile to add a bit of information on the
difference between frequentist and Bayesian approaches (as the latter do allow to incorporate uncertainty in source isotopic composition to a degree, be it temporal or spatial), and the
development of the field as such.

We added a paragraph in introduction between the two approaches from lines 93 to 105. “There are two main approaches to modelling in mixing models. Historical models typically use a frequentist approach [7, 13, 37-40], which provides single-value estimates along with confidence intervals. Recent developments have shifted towards Bayesian approaches [17-19], such as MixSIAR, which offer probability distributions of estimates rather than point values. This Bayesian framework represents a significant advancement, particularly in its ability to manage uncertainties and errors in data, parameter estimations, and statistics [41, 42]. However, Bayesian models do not facilitate model evaluation [43]. Furthermore, despite these improvements, existing Bayesian mixing models still do not incorporate λ, the isotopic turnover rate, which is crucial for accurately accounting for temporal dynamics in diet studies. Although these models handle source variability to some extent and work similarly to static models (SMMΔ), they remain limited in their ability to fully capture the dynamic nature of isotopic incorporation. Notably, no current Bayesian MM can integrate the full range of temporal isotopic dynamics—particularly the role of λ—as proposed in this study.”

The Methods and results are very detailed and contain all information necessary to follow the later discussion, maybe even too much information at times. Some paragraphs in the Methods
section read a bit like a mixture of introduction and Methods (I´ve indicated sections where I thought this to be the case below).

We took off parts in material and methods that read too much like an introduction to go straight to the point (see response for comments below).

Similarly, the Results sometimes interpret the data to an extent that I would expect more for a discussion (also indicated below). The discussion of this paper is very long and could benefit from more focus at places. It also should put the results of the study more into context with the existing literature. As it stands, the discussion largely discusses the validity of the models used, and repeats results that were already shown in the results section. The paper of Phillips et al. (2014) is heavily cited in the discussion, but other literature on mixing models (e.g., Stock & Semmens; Canseco et al.)
is not discussed.

We thank reviewer 1 for the comment, we discussed Stock and Semmens line 720 and 729-732. However, the only articles we found of Canseco et al. discuss TDF matters that we found out of the scope of this study.

Overall, I think the discussion could benefit from more structured, streamlined writing with a clearer focus on the relevant results and what they mean for the field.

We have already addressed this point in the first comment. For the focus on the results and the link to the field see later comment.

All figures of this paper need a thorough revision! A figure caption should be able to stand on its own and state clearly what data was used for the figure and how it has to be interpreted.

Figures were revised. Figures 3 to 9 were changed to fit to reviewer 2’s comment. Captions were changed to clearer understandability.

- Line 213-216, Fig 1: From “Framework and operation for the SMM, SMMΔ and DMM (see Table 1 for definitions and acronyms).” We added line 214-216: “The processes for the three models are depicted with dark lines, showing the required inputs, the diet estimation time (or period, depending on the model), and the process or solution selection. i and i+1 represent two sampling dates.”
- Line 283-286 was changed from: “Fig 2: in-silico process to estimate the bias. The complete lines represent the use of a mixing model. The in-silico framework boxes express what is used as data in the following mixing models (see Fig. 1).” To: “Fig. 2: In silico process for estimating the bias. The solid lines represent the use of a mixing model, and the framework boxes indicate the data used in the corresponding mixing models (see Fig. 1). Each box represents a step in the bias estimation process, and the dashed arrows show the data used at each step and the order in which it is applied.”
- Line 298-300, Fig 3: Experimental setup in-silico. The figure was changed a little bit the number of tested changed from twenty to three. The number of diets were changed from 3 to 4. The name of the figure changed to “Fig. 3: In silico experimental setup.” And we added the following caption “Each column represents a distinct experiment, showing the effect being tested and the number of modalities used, such as the number of tested diets, the λT value tested, the number of consumer initial values and the source variation scenarios.”
- Line 407-413: We changed the figure title from Fig. 4: Diet switch effect on bias for diet 1. To “Diet switch effect on bias for a single-source diet for constrained (diet 1, 100% of source 3) and unconstrained systems (diet 4, 100% of source 3).” and added the following: “The first row shows the results for diet 1 (A and C), and the second row shows the results for diet 4 (B and D). The dots in Panels A and B represent the median bias values for all the solutions within a 0.5‰ tolerance. The crosses represent the quartiles. Each colour corresponds to an initial value and its distance from equilibrium (switch percentage). These are shown in the source polygons in Panels C and D, where the triangle indicates the equilibrium signature.
- Line 428-435, the title from Fig. 5 was changed from “Fig 5: Diet switch effect on bias for diet 2 and 3.” To “Fig. 5: Effects of diet switching on bias for mixed diets for constrained (diet 2, 15% of source 1, 45% of source 2 and 40% of source 3) and unconstrained (diet 3, 20% of source 1, 20% of source 2, 40% of source 3 and 20% of source 4) systems. “. We changed the caption from “(A)Diet Switch effect (ie. Consumer’s initial value) on bias as a function of λT for the second scenario: 3 sources, 2 isotopes, with a diet consisting of 15% of source 1, 45% of source 2 and 40% of source 3. (B) The source polygon is shown with the equilibrium signature represented by a triangle (15% of source 1, 45% of source 2 and 40% of source 3). (C) Diet Switch effect on bias as a function of λT for the third scenario: 4 sources, 2 isotopes, with a diet consisting of 20% of source 1, 20% of source 2, 40% of source 3 and 20% of source 4. (D) The source polygon is shown with the equilibrium signature represented by a triangle (20% of source 1, 20% of source 2, 40% of source 3 and 20% of source 4). The initial values are coloured according to its Euclidian distance to equilibrium.” to “The first row shows the results for diet 2 (A and C), and the second row shows the results for diet 3 (B and D). The dots in Panels A and B represent the median bias values for all the solutions within a 0.5‰ tolerance. The crosses represent the quartiles. Each colour corresponds to an initial value and its distance from equilibrium (switch percentage). These are shown in the source polygons in Panels C and D, where the triangle indicates the equilibrium signature.”
- Line 443-450: We changed the title of the figure from Fig 6: Source effect bias as a function of λT for four different pre-switch consumer values in the first scenario (three sources, diet 100% of source 3) to “Fig. 6: Effect of source variability on bias as a function of λT for three initial consumer values in diet 1 (with three sources, 100% of the diet from source 3). and modified the caption from “This scenario considers varying isotopic values of the sources. The coloured points represent the bias when the sources are stable over time (therefore identical for SMM and SMMΔ cf Fig. 4), while the grey area represents the additional bias with dynamic sources over time. (A) Bias for a low distance initial value. (B) Bias for a medium-low distance initial value. (C) Bias for a medium-high distance value. D) Bias for a high distance value”

to : “The median biases are represented for three consumer initial values: one close to equilibrium (A, 0% switch), one in the middle of the polygon (B, 50% switch), and one far from equilibrium (C, 100% switch). For more details, see Fig. 4C. The coloured points represent the median bias value in the source-constant scenario for solutions falling within a 0.5‰ tolerance. The grey envelopes represent the maximum and minimum median biases in the dynamic source scenarios for the top 5% of the best solutions.

- Line 467-471. We changed the figure title from “in-silico environment with an example of a set of 10 sampled dates” to: “Fig. 7: Carbon (A) and nitrogen (B) simulated signatures over time in the in silico environment and an example of 10 sampled consumer signatures.”. We then added A and B on the figure and the following caption: “The blue line represents the simulated complete time series of the consumer’s isotopic signatures over time. The red lines are the simulated complete time series of the sources’ isotopic signatures over time. The black dots are examples of 10 sampled values used for diet 1 simulation.
- Line 472-474: The figure changed and is now a boxplot. We changed the title from “Bias of the three mixing models to estimate the reliability of DMM” to : “Fig. 8: Boxplot of median biases for the three models relative to the field-sampled-like dataset.” We added the following caption: “The blue line represents the simulated complete time series of the consumer’s isotopic signatures over time. The red lines are the simulated complete time series of the sources’ isotopic signatures over time. The black dots are examples of 10 sampled values used for diet 1 simulation.”
- Line 493-497: we changed the title of the figure 9 from “Estimated diet contribution (50 best solutions) of each food sources over time for the geese dataset.” To “Fig. 9: Comparison of source contribution estimations over time by the SMM and DMM in the goose dataset and bias estimations”. We also changed the caption from “SMM (A) and DMM (B) outputs. The estimated bias on the best solutions for the SMM compared to the DMM used as a reference is shown in (C) where numbers represents the sampling date. Each bias is coloured according by the distance to the equilibrium (estimated with the DMM results)”

to “ Panels (A) and (B) show the contributions of each food source over time, estimated by the SMM and DMM, respectively. The lines represent the medians, and the envelopes represent the quartiles. Panel (C) displays the median bias according to λT, with the median DMM estimations used as the reference diet.”

- Line 544-548 we changed the title of figure 10 from : “Fig 10: Decision tree to help choose for the most suitable model. Adapted from Ballutaud et al.” to “Fig. 10: Decision tree for selecting the most suitable model among the SMM, SMMΔ, and DMM. we added to figure 10 caption: “Adapted from Ballutaud et al.[13]. Each circle represents a key aspect of the system that should be considered when selecting a mixing model, guiding the choice among the three models. *The use of any MM in the λT> 2 condition may lead to a high bias depending on the system and its dynamics..”

I´ve included some more detailed comments below.
Specific comments:
L15: "quantify" instead of "decipher"?

L15 done
L16: Maybe just write "These models assume", as they are STILL based on that assumption, not only historical (most mixing model studies still use SMMs, no?)

L15-16 Corrected by “Most of these models assume”
L21: relatively to what? Maybe delete?

done
L26: "isotopic integration"? Do you mean turnover?

Changed by line 25 “time variability linked to isotopic turnover”
L27: Maybe write "dietary changes" instead of "diet switch"? Could also be caused by changes in food availability than an active switching of sources.

Changed by line 26: “dietary shift”
L63: Stock & Semmens 2016 would also be an important paper to introduce here (unifying error structures to estimate variation)

This article was introduced line 98 while introducing error structure and then discussed in discussion (line720 and 729-732) when speaking about error structure and Bayesian.
L117-120: This section reads like an intro. Maybe introduce the model classes needed for your study in the intro and focus the Methods on a simpler structure of "to achieve X, we did Y"?

This section was removed. It was replaced as line 135: “The static mixing model (SMM) is based…”
L131-134: See above, this is a lot of details on the "why" of this model, which a reader should already know after reading the intro.

This paragraph was removed and changed line 150 by “To address limitations identified by Phillips et al….”
L202-206: Is all this intro really needed to understand how you estimated bias?

We changed the definition from: “In a studied system with *S* different sources, a consumer has a real diet composed of specific proportions from each source (the contribution of the source *i* to the real diet is denoted as $p_{i}$ and $\sum_{i=1}^{S} p_{i}=1$). When applying mixing models, we estimate this diet and obtain an estimated proportion for each of the *S* sources consumed (the estimated contribution of the source *i* to the diet is called $\hat{p_{i}}$). The bias for a single source is defined as the difference between its real contribution to the diet and its estimated contribution by the model” to line 235-241: “The bias for a single source (denoted as i in a system with S sources) is defined as the difference between its actual contribution to the diet (denoted $p_{i}$) and its estimated or predicted contribution by the model (denoted $\hat{p_{i}}$). To estimate the overall system bias, denoted as β, we sum the absolute value of the bias for each source (Eq. 7). This bias ranges between 0 and 2, where 0 indicates no bias (i.e., the model accurately estimates the real diet) and 2 indicates complete bias (i.e., the model fails entirely to estimate the real diet).

Also, the bias expression (Eq.7) were changed following a comment from reviewer 2 (line 243).
L240-241: What do you mean by constrained here? No more than x tracer + 1 sources? Please clarify.

We added some precisions lines 262-264: constrained system “with n tracers for n+1 source” and unconstrained system “with more than n+1 sources for n tracers”

L322: "was then simulated"

Sentence changed line 353-355 by “This setup provided a complete simulated dataset representing the potential dynamics of an isotopic system in the environment”
L322-323: Most studies approach their study system with some prior knowledge of the seasonal dynamics. Were those 10 sampling dates chosen randomly, or did you set them at equal intervals?

Sentence changed line 357-358 to “Consumer signatures were sampled on 10 distinct dates, chosen randomly without regard to seasonality or the interval between samplings.”
L417-418: Sounds like a discussion point

Line 419-420 removed from results and added line 536-538 “At high λT values in complex and dynamic systems, all three models show high variability in bias, which can be partially attributed to the complexity of the system itself.”
L421: Why? Point of discussion, why is this the key observation?

The sentence was changed line 458-459: from “The key observation lies in the region where λT <2. “ to “A significant difference in bias between the models was observed in the λT < 2 region”
L476-479: This is repeating your results, I would remove the figure references and just discuss the interpretation without repeating the results leading up to it.

We removed the figure reference and only let the interpretation. The text changed line522-524:” the DMM exhibits minimal bias, whereas static models become completely biased, making the DMM the most reliable deterministic mixing model available.”
L480-483: Not sure this is the right place to discuss the limitations. Rather put your findings into context with other studies.

This limitation paragraph was moved on a new part named “DMM limitations” and changed to line 669-673: “Additionally, MMs provide only an average diet over the time window under study. When applied over long time windows (depending on the associated λ value, e.g., with annual sampling for large species or monthly sampling for larvae with high λ [52]), this can result in reduced precision and potential bias.”

Some context to other studies was added line 615 to 635 (see comment next page).
L484-498: Possibly outside of the scope of this study, but I would be interested how a Bayesian MM would compare in the unconstrained cases?

As we changed the number of kept solutions, it smoothed the patterns observed for un-constrained cases making them similar to constrained patterns.
L491-498: This section describing Phillips et al. recommendations on mixing polygons could maybe be summarized in one or two sentences to start off the next paragraph?

We changed this paragraph from: “Philips et al.[17] recommended ensuring that the consumer signatures remains within the range of the source signatures. Visually, this means that the consumer signature should fall within a polygon defined by the source signatures. When this condition is unmet, it indicates that the source polygon shape has shifted since the previous equilibrium and that the consumer signature is still transitioning toward a new equilibrium. Since static models assume equilibrium as a foundational principle [2], they become invalid in such scenarios. Verifying the source polygon ensures that the consumer is relatively close to equilibrium, supporting more reliable model applications.”

To line 640-645: “This issue is particularly relevant to Phillips et al.'s recommendation that consumer signatures should remain within the range of the source signatures. If this condition is not met, it suggests that the source polygon shape has shifted and that the system—especially the consumer—is not at equilibrium. Under such circumstances, a static mixing model assuming equilibrium [8] should not be applied.”

This paragraph was moved from its previous position and is now logically connected to the following paragraph discussing adherence of these recommendations in literature.

L505-515: Again, multiple repetitions of results in this section, which I don´t think are strictly necessary to understand the paper and inflate the length.

This part was removed.
L516: I stopped commenting every repetition or reference to specific sections of the results after this one. My recommendation would be to remove most, if not all from your discussion.

- Line 536-538: sentence changed from “At high λT values (Fig 8), the bias across all three models exhibits high variability ranging from 0% to 50%.” To “At high λT values in complex and dynamic systems, all three models show high variability in bias, which can be partially attributed to the complexity of the system itself.” The next sentence was changed as well to fit: line 539: we added “to temporal patterns in diets such as” before “sinusoidal contributions and line 540 we added “in this study”.
- Line 524 reference removed The sentence was changed from “. This is shown with the in-silico environment (~~Fig. 4-6~~) “ to “The in silico environment used in this analysis demonstrates the robustness of the DMM”
- Line 578 reference removed “all models, including dynamic ones, may exhibit increased bias ~~(Fig. 7–8).”~~

L517-518: Outputs commonly observed, but you offer no examples. I would recommend to use way more literature to put your results into context, you already have a lot from your review, no?

We thank reviewer 1 for this comment. This sentence was removed from the manuscript

We added line 615-635 two paragraphs to put some context in our results here:

“. One scenario involves studies where multiple source and consumer signatures are already collected over time. This is often the case in studies investigating seasonal effects, where one signature is sampled per season [29, 61], or in aquaculture studies tracking diet changes in growing animals with one sampling per growth period [62]. Some studies also sample prey signatures over a period related to λ before measuring consumer signatures [28, 63-65]. These studies, which already account for temporal dynamics, could readily adopt the DMM for more accurate estimations.

Another scenario involves studies that initially treat their data as static but, in fact, include multiple sampling points over time. These studies could transition to a temporal framework with minimal adjustments. The most straightforward case occurs when repeated sampling is conducted over time for both consumer and source signatures [66-69]. These datasets can directly support temporal analysis. In other cases, where only one of either the consumer or source signatures is sampled over time, the DMM can still be applied with minimal additional data. For example, Johnson et al. [70] presented multiple consumer signatures over time and attempted to estimate dietary changes with only a single estimation of source signatures. By adding a second sampling of source signatures, the study could complete a temporal survey. Similarly, studies employing opportunistic sampling, such as those by Gama et al. [71], Ogilvy et al. [72], and Rosas-Luis et al. [73], could benefit from a temporal approach, as they incorporate long-term consumer data. Although uncertainties might arise owing to inconsistent sample sizes in opportunistic studies, the DMM can help estimate diets by inferring source and consumer signatures over time. “

This paragraph also included the adding of 13 new citations coming from the first appendix to help put results into context.

61.	Kundu GK, Kim C, Kim D, Bibi R, Kim H, Kang C-K. Phytoplankton fuel fish food webs in a low-turbidity temperate coastal embayment: A stable isotope approach. Frontiers in Marine Science. 2021;8:751551.

62.	de Moraes CRF, de Attayde JL, Henry-Silva GG. Stable isotopes of C and N as dietary indicators of Nile tilapia (*Oreochromis niloticus*) cultivated in net cages in a tropical reservoir. Aquaculture Reports. 2020;18:100458.

63.	Catry T, Lourenço PM, Lopes RJ, Carneiro C, Alves JA, Costa J, et al. Structure and functioning of intertidal food webs along an avian flyway: a comparative approach using stable isotopes. Functional Ecology. 2016;30(3):468-78.

64.	Catry I, Sampaio A, Silva MC, Moreira F, Franco AM, Catry T. Combining stable isotope analysis and conventional techniques to improve knowledge of the diet of the European Roller *Coracias garrulus*. Ibis. 2019;161(2):272-85.

65.	Dromard CR, Bouchon-Navaro Y, Harmelin-Vivien M, Bouchon C. Diversity of trophic niches among herbivorous fishes on a Caribbean reef (Guadeloupe, Lesser Antilles), evidenced by stable isotope and gut content analyses. Journal of Sea Research. 2015;95:124-31.

66.	McClain‐Counts JP, Demopoulos AW, Ross SW. Trophic structure of mesopelagic fishes in the Gulf of Mexico revealed by gut content and stable isotope analyses. Marine Ecology. 2017;38(4):e12449.

67.	Nash LN, Kratina P, Recalde FC, Jones JI, Izzo T, Romero GQ. Tropical and temperate differences in the trophic structure and aquatic prey use of riparian predators. Ecology Letters. 2023;26(12):2122-34.

68.	Prado P, Baeta M, Mestre E, Solis MA, Sanhauja I, Gairin I, et al. Trophic role and predatory interactions between the blue crab, *Callinectes sapidus*, and native species in open waters of the Ebro Delta. Estuarine, Coastal and Shelf Science. 2024;298:108638.

69.	Robinson BG, Franke A, Derocher AE. Stable isotope mixing models fail to estimate the diet of an avian predator. The Auk: Ornithological Advances. 2018;135(1):60-70.

70.	Johnson DL, Henderson MT, Anderson DL, Booms TL, Williams CT. Bayesian stable isotope mixing models effectively characterize the diet of an Arctic raptor. Journal of Animal Ecology. 2020;89(12):2972-85.

71.	Gama LR, Fuentes MM, Trevizani TH, Pellizzari F, Lemons GE, Seminoff JA, et al. Trophic ecology of juvenile green turtles in the Southwestern Atlantic Ocean: insights from stable isotope analysis and niche modelling. Marine Ecology Progress Series. 2021;678:139-52.

72.	Ogilvy C, Constantine R, Bury SJ, Carroll EL. Diet variation in a critically endangered marine predator revealed with stable isotope analysis. Royal Society Open Science. 2022;9(8):220470.

73.	Rosas-Luis R, Navarro J, Loor-Andrade P, Forero MG. Feeding ecology and trophic relationships of pelagic sharks and billfishes coexisting in the central eastern Pacific Ocean. Marine Ecology Progress Series. 2017;573:191-201.

L552: Maybe write out the parameter so readers that may have forgotten what lamda T means can still make something of the header

Line 557-558 Header changed by “Importance of considering isotopic turnover and sampling/experiment time (λT) in isotopic studies”
L616-627: This whole section feels reads like a repetition of your intro. I would recommend to either use the review for the intro (which works well I think) OR the discussion, not both.

We thank reviewer 1 for their comment and agree that the portion they highlighted is too much alike the introduction. However, we feel that we tried to convey different ideas using the review than in the introduction and that it is relevant in the discussion. We changed these paragraphs so that they do not repeat themselves from the introduction.

- line 611: When showing that 25% of reviewed article could theoretically do dynamic studies, we added “Theoretically” to the sentence

- line 614: The rest of the paragraph is a repetition of the introduction and was removed. The part removed stated: “Despite being a founding hypothesis for isotopic analysis, system stability is rarely looked upon in literature. In the corpus, only 25% of the articles accounted for stability or instability of sources (either by assuming or verifying the hypothesis) and only 18% conducted temporal sampling of sources signatures. This leaves 75% of studies operating under the assumption of "steady state" system without verifying this premise, potentially introducing significant bias into their static mixing model estimations. This oversight is concerning, as variations in source and consumer signatures are quite common [23, 38, 51, 61, 62]. These findings emphasize the critical importance of assessing and accounting for source dynamics in isotopic studies.” Which is very like the intro.

- The citations that were removed were added in the intro line 87 within the sentence “Although this assumption may be valid in some cases, temporal changes in isotopic components—such as seasonal baseline shifts—have been observed [24-30].”

Mir-Arguimbau J, Navarro J, Balcells M, Martín P, Sabatés A. Feeding ecology of blue whiting (*Micromesistius poutassou*) in the NW Mediterranean: the important role of Myctophidae. Deep Sea Research Part I: Oceanographic Research Papers. 2020;166:103404.

D'Ambra I, Graham WM, Carmichael RH, Hernandez Jr FJ. Dietary overlap between jellyfish and forage fish in the northern Gulf of Mexico. Marine Ecology Progress Series. 2018;587:31-40.

Lloret-Lloret E, Navarro J, Giménez J, López N, Albo-Puigserver M, Pennino MG, et al. The seasonal distribution of a highly commercial fish is related to ontogenetic changes in its feeding strategy. Frontiers in Marine Science. 2020;7:566686.

Meyhoff SD, Johnson DL, Ellert BH, Lutes K. Seasonal changes of stable isotope signals in the primary feathers of plains sharp‐tailed grouse. Wildlife Society Bulletin. 2023;47(2):e1412.

-The paragraphs line 646-662 were modified to more clearly presenting other statement than the intro. We removed the sentences line 603 to 608 “For instance, while 87% of the studies carefully selected TDFs, only 29% followed the recommendation to verify if the consumer's isotopic signature falls within the source polygon. As exposed earlier, temporal effects, a key source of potential bias, were considered in just 18% of the studies. Furthermore, λ, a crucial parameter for minimizing bias, was acknowledged in only 26% of the articles and used to guide adapted sampling protocols in a mere 4%.”.

We then replaced it by the following statement line 650-658:” Specifically, we selected three key guidelines for evaluation: (1) whether the authors carefully selected trophic discrimination factors (TDFs), (2) whether they ensured that their consumer’s isotopic signature fell within the source polygon, and (3) whether temporal sampling for the sources was conducted. We found that 5% of the articles followed none of these guidelines, 58% followed only one (usually the choice of TDF), 34% followed two (typically TDF selection and checking the source polygon), and only 6% followed all three guidelines.

Furthermore, as previously noted, only 26% of the articles discussed λ, a crucial parameter for minimizing bias.”
L630-639: Why is this in conclusions, or rather, WHAT is the conclusion here?

- Indeed, this paragraph seems a little unclear about its conclusion so we reshaped it. We removed the part line 645. “In scenarios where the actual diet is known- such as controlled or semi-controlled conditions, and in-silico frameworks- it helps identify conditions where mixing models produce errors. By cataloguing these conditions and their associated bias, corrections can be developed to enhance model accuracy. Identifying specific scenarios prone to bias allows to estimate potential biases in static mixing models applied to in-situ data through exploratory analysis. For instance, observing a field-collected data with significant consumer signatures fluctuation over time can reliably indicate the likelihood of biased estimates for both SMM and SMMΔ compared to DMM. Additionally, bias studies may reveal additional factors not addressed in this study.”
- We then replaced it by line 689-693: “This serves two main purposes for readers. First, it alerts researchers to the use of mixing models to estimate the potential for significant bias in diets and underscores the importance of accounting for it. Second, it helps them recognize common scenarios that lead to high bias, enabling them to address these issues in their datasets, sampling plans, and analyses. Bias studies such as this one may reveal additional factors not addressed here

L640-646: I think this section (barring the repetition of results at the end) would be a better leading paragraphs for the conclusions

The first two paragraphs of the discussion were switched.

The repetition of results (line 692) was removed.

The two first paragraph of the conclusion were joined as they are in continuity. We removed line 693 to change it as a “for example”.

L658-661: I think this lifetime perspective of mixing models would ONLY work if an all individuals can use the same resources along their lifetime. For gape-limited predators, a mixing
model over their lifetime would assume a juvenile (e.g., a newborn shark <50 cm) could, in theory, experience a contribution from the sources of an adult (i.e., very large prey items)

It can work as well with consumer changing food source over time as you can change potential food sources in the model depending on the age of the consumer.

We added the sentence line 706-707 “It also necessitates temporal monitoring of potential food sources that may change over time.”

L667-672: Yes, but you didn´t assess variation in TDFs. This conclusion paragraph appears rather chaotic and unfocussed. I think it would be best to split it into a limitations paragraph,
in which you could discuss the limitations of YOUR models along with the things you didn´t look at, and a conclusions paragraph that specifically highlights the take home messages of
your study and outlines future directions.

- This paragraph was moved from the conclusion to the added section “DMM limitations” (line 674-679). We added the following part as well line 679-684: “Although this study did not focus on TDF values or their dynamics, which could introduce an additional layer of bias beyond what was examined here, it is important to note that this would create an even more complex system where identifying bias becomes more challenging. The code used for modelling the DMM was developed with the concept of a dynamic TDF in mind. Users have the option to apply the DMM with a time-varying TDF to account for this additional complexity.”

Appendix 6: Maybe reiterate what were the simulated diets/isotopic systems.

Systems were described again in Appendix 6
Appendix 7: How do I see which space of the six isotopic spaces is which from the figure A, in order to reference it to figure B?

The previous Appendix 7 is now Appendix 2.

We associated each space with a color that is used then in the second figure estimating bias for more clarity. We also added a table exposing the size of space tested.

Reviewer #2:

General remarks

The manuscript aims to describe and analyze bias in mixing models caused by temporal

dynamics and to develop a dynamic mixing model. The authors also discuss the influence of the

isotopic turnover rate and sampling frequency on the accuracy of mixing models both under the

steady-state hypothesis and in the dynamical approach. The manuscript's focal point is the

discussion of biases arising due to the absence of temporal dynamics in said models. This is an

interesting topic belonging to a broader range of problems related to the estimation of biases

and uncertainties in the modelling of stable isotope mixing, rarely discussed in the literature in

sufficient detail. As the SMM calculations typically involve an underdetermined system, i.e. the number of potential food sources typically exceeds the number of measured isotopic signatures, the

careful consideration of correlations and uncertainties is crucial and remains a challenging topic

both conceptually and in terms of computational complexity. The resulting probability

distributions of fractions describing contributions to the mixture from various sources are usually

broad and highly correlated, thus preventing drawing any conclusions from central values (mean

or median) alone. Additionally, particularly in the studies of trophic networks, there are

numerous processes influencing the isotopic composition of tissues (e.g.: trophic discrimination

factor, isotopic turnover rate or element concentrations), which results in a high number of

model parameters, further increasing the calculation complexity of computation and error

estimation.

Following the above consideration, in my view, the manuscript contains several serious flaws

that undermine its conclusions, making it unsuitable for publication in a journal. My concerns

with the presented methodology primarily relate to the bias calculation method and, more

importantly, the implementation of mixing models, which fails to account for correlations. This

omission results in an incomplete and potentially misleading discussion of systematic

uncertainties.

We thank reviewer 2 for their detailed feedback. We appreciate the reviewer’s recognition of the importance of the subject matter, which we believe addresses a crucial yet often overlooked aspect of isotope ecology. We strongly agree that understanding and quantifying bias in mixing models is fundamental to improving the robustness of conclusions drawn from isotopic data.

Regarding their specific concerns about the bias calculation method and the implementation of mixing models, we have carefully considered their specific comments below. In response, we have revisited our methodology to clarify the treatment of correlations and to improve the accuracy of the bias estimation.

Regarding the manuscript’s readability, it requires significant improvement. The structure feels

chaotic and the text is often unclear, with issues in grammar and typos that disrupt readability.

To enhance clarity and coherence, the authors should refine the language, avoid technical

jargon, improve the logical flow, and ensure proper adherence to typographical standards. A

thorough revision is needed to achieve acceptable presentation standards.

Based on all these factors, it is impossible for me to approve this manuscript. I should however

state that the topic of biases in mixing models is very much a worthy pursuit and I encourage

the authors to not abandon the topic and try to repeat the analyses with refined methodology.

Below, I outline key criticisms and suggestions for improvement in the hope that they will not

discourage the authors but rather contribute to a deeper understanding of the subject.

Following similar comments from Reviewer 1 we have improved the clarity and structure of the overall manuscript (see reviewer 1 comments), and in particular of the discussion section, see the comments above. The grammar was reviewed by Springer Nature Author Services (see on SNAS website with the verification code 5891-6FC4-78A8-FBFF-C759 or the attached certificate).

Criticism

Introductory section

The article makes a case that the majority of studies involving SIMMs published in 2014-2024

do not address the potential temporal changes in analyzed diets. The introductory section

leaves the reader with the impression that these studies are faulty and the wrong assumption is

made about the steady state of the system. This is a major criticism towards 75% of 103

published papers and in my opinion, this criticism is too strong and not motivated well enough in

the manuscript. The steady-state assumption may be an accurate approximation for a given

system and thus the temporal dependence is not simply overlooked. In the current formulation,

the manuscript appears to undermine the competence of many authors (which is not a problem

in itself, but then it requires much more information).

The authors thank reviewer 2 for their feedback. We appreciate the concern, and we understand how the way we presented the critique could be interpreted as overly harsh. Our intent was not to undermine the competence of the authors whose studies we reviewed, but rather to point out that many of the papers implicitly assumed a steady-state system without explicitly verifying or accounting for potential temporal changes.

We have made the following revisions to clarify our position:

- We changed the expression line 76 “made effort to estimate” to “actively estimated”
- We clarified what type of article we selected by modifying the sentence line 73-74: “we reviewed 103 scientific articles that used mixing models to estimate diets”
- Added “temporal” line 83 and “or verified the system’s stability” line 83
- Added the sentence “Although this assumption may be valid in some cases” line 85-86
- Line 85 We added “Implicitly”

Our aim was simply to highlight that a significant proportion of reviewed studies did not explicitly state the steady-state assumption while using models that implied it. In those cases, if the system was dynamic, diet estimations might have been biased. We did not intend to criticize the authors but rather to point out an overlooked aspect that could lead to biases, which we felt was important to mention. We hope this clarifies our position and removes any judgmental tone that may have been perceived.

Methodology - calculation of bias

I do not find the method of bias calculation presented in the manuscript sufficiently accurate.

The definition of the 𝛽𝑆 factors in Eq. 7 that excludes a single source S is justified with the

remark that “calculating bias for every source in the system simultaneously [...] the biases would

sum to zero“, which I cannot agree with. While the statement is technically true, I see no reason

for selecting such a definition instead of 𝛽i = 𝑝𝑖 - 𝑝̂i. Using this definition, the number of 𝛽

parameters remains the same, while at the same time, we obtain information on the

directionality of the bias for each source individually. Alternatively, if an aggregate bias for the

whole system is of interest, one could sum the absolute values or the squares of the 𝑝𝑖 - 𝑝̂i, this

way avoiding the trivial zero-sum. The definition of the 𝛽𝑆 factors used in the manuscript is

confusing and difficult to interpret.

In my opinion, the calculation of bias must account for the width of the probability distribution of

the solutions to the mixing problem. The significance of the bias can only be inferred when

compared to the width of such distribution.

The authors thank reviewer 2 for their insightful comment regarding the bias calculation. Indeed, estimating the bias of each source individually and summing the absolute value to obtain the general bias of the system is a simpler approach. We have implemented this definition of Bias in the revised manuscript as per the reviewer’s suggestion.

The bias is now defined between 0 and 2, compared to the old version, which defined it between 0 and 1. As the sum of the proportions still equals 1, the bias value for 1 source is the same as the cumulative bias for all other sources. The updated bias calculation changed our results, as all previously observed values are now multiplied by two, due to the addition of the bias from the last source. However, although we agree this is a simpler and more intuitive calculation, this change does not affect the observed trends or the relationships between bias and λT values, nor does it alter the conclusions drawn from those trends.

Line 37: $\beta_{S}$ changed by $\beta$ “Relative system bias”

Line 238-239: modification of the definition of bias: “To estimate the overall system bias, denoted as Bias, we sum the absolute value of the bias for each source (Eq. 7).

Line 239-240: This bias ranges from 0 to two so we corrected the values: “This bias ranges from 0 to 2…”

Line 243: Eq.7 y:$\beta s=\sum_{i=1}^{S-1} \left| p_{i}-\hat{p_{i}} \right|$ was replaced by:$Bias=\sum_{i=1}^{S} \left| p_{i}-\hat{p_{i}} \right|$

Line 244: We removed equation 8 since it does not work anymore with the new definition of bias. We also removed the paragraph explaining it.

Line 244-245: We introduced the relative bias of the system β: “For the remainder of this study, the bias is expressed as the relative bias of the system β expressed in % of the maximal bias (see Eq. 8).”

Line 246: We added Equation 8: $\beta=\frac{Bias}{2}100$

Line 248-249: we changed the sentence from “This equation highlights three key parameters that influence the bias in the SMM model” to “When the time model (Eq. 3) is used to model isotopic integration, three key parameters influence the bias in SMM estimates”

Line 281-282: correction from “bias values were calculated on source 3 (β_3),…” to “bias values were calculated following the methodology outlined in Equation (7) and Equation (8)”

Line 367-368: We changed the sentence “the bias of the applied DMM (using only the sampled data) was computed ~~on source 3~~.”

Line 392: We removed the sentence “The bias was quantified for the aggregated UlvaxEnteromorpha source by comparing the best solutions generated by each model.”

Also, all figure representing bias (Figures 4,5,6,8,9) as well as figures in S6 Appendix we redone to the right bias definition. They were also redone in order to present statistical uncertainty (see two comments belows)

Methodology - implementation of mixing models

The implementation of the mixing models uses a so-called “brute force” as finding the solution

requires generating all potential combinations of source contributions (with user-defined

precision). The computational complexity thus scales with the power of the dimensionality of the

problem, which for the typical real-world use cases may rapidly explode.

This is a very interesting comment as the running time may be quite long for complex mixing models (Ex. MixSIAR). In fact, computational complexity of our models is moderate and take shorter execution time as Bayseian models as it is. As an example, the code used to run the in-silico experiments on bias (all three models executed on 20 λT conditions, 5 different source variation scenarios and 3 initial value, in other words for 300 iterations each) runs for less than ten minutes, meaning that when using it for diet reconstruction studies, if the precision is not excessive, the model runs quite quickly. Also, running deterministic mixing model as our own is much shorter than running MCMC chains.

However, a significantly greater concern comes from the fact that the correlations are disregarded completely and these are absolutely crucial in any discussions involving uncertainties of the mixing models.

As already stated, the probability distributions of fractions describing the solutions to the mixture

from various sources are usually broad, skewed and highly correlated. The central values alone

cannot provide sufficient information to draw conclusions regarding any type of uncertainty (both

statistical and systematic). Additionally, as mentioned in the previous paragraph the bias should

be studied in relation to the statistical uncertainty, which is completely absent in presented

studies.

It is right that correlations were partially disregarded in the first version of the MS . These correlations occur as the sum of contribution of every source equal to 1, every value depending on the others to a point. However, with the new bias definition given by reviewer 2 (see two comments above), with the general bias summing error for every source contribution estimation, this absorbs the additional error that the correlation could bring on separate bias on each source. Additionally, source correlation appears when the model fails to discriminate between two sources. In our experimental framework, both source and consumer values were chosen to avoid correlation.

Also, the correlations were taken into account in the geese case study as we aggregated two correlated sources see lines 389-391: “To address the high correlation between the two dietary sources, Ulva lactuca and Enteromorpha spp., we combined them into a single source (Ulva x Enteromorpha), as recommended by Phillips et al.[22].”

We agree that statistical uncertainty is an important part of in models. We changed every model calculations and representation from presenting the best value given by the model to the median, first quartile and last quartile for a selection of solution. The best kept solutions were either the 5% best solutions out of every combinations of possible solutions or all solutions that fit the observation under a specific tolerance.

- In materials and method, we added precision in the model functioning presentation: lines 202-205: “Afterwards, the user can choose to either select a subset of combinations that best fit the observed consumer signatures or retain all combinations that predict a consumer signature within a certain tolerance of the observed value.”
- Lines 278-281: we added to the sentence: “Both SMM and SMMΔ were subsequently used to estimate the contributions of diet. For each model, either the top 5% of best-fitting solutions were retained, or all solutions that fit the data within a 0.5‰ tolerance were retained.”
- In material and methods we refined the mode of solution selection Line 309:” For each case, all solutions yielding estimates within a 0.5‰ tolerance were retained
- Same thing line 318-320: “In constant-source scenarios, where exact solutions were produced, all solutions yielding estimates within a 0.5‰ tolerance were retained. In scenarios with dynamic sources, the top 5% of best-fitting solutions were retained.”
- Line 387-388: we specified how the solutions were kept:” The model was set to retain the top 1% of the best solutions (50 solutions) with a generated precision increment of 0.01‰.”
- Same line 409-411: “The dots in Panels A and B represent the median bias values for all the solutions within a 0.5‰ tolerance.”

Keeping a number of solutions brought also improvement in our results especially regarding un-constrained cases. Keeping the median and quartile values gave smoother results (see figure 4 and 5) resembling more of the constrained case and strengthening our claim about the link between bias and λT. To confront constrained vs unconstrained scenarios while staying clear enough, we added a 4^th^ studied diet in an unconstrained system and reduced our studied initial values to 3 for both experiments

- Line 272-273: We added “4. Un-constrained system, single source diet: the real diet is composed solely of source 3.”
- Line 266: three was changed to “four”
- Line 305 : 20 consumer values were changed by “three”
- Line 314: Four consumer value changed to “three” and added line 314-315 “(same as the switch effect values)”
- Line 321: three diet scenarios was changed to “four diet scenarios”
- Line 298: We changed figure 3 to it indicates the proper number of diets/ scenarios

Accordingly, we changed Figures 4,5,6,8,9 and appendix 5,6,7 so they present median (and the first and last quartile) of estimated bias with all kept solutions. The captions of figures 4,5,6 were changed to best describe the graph (see comment of reviewer 1 about figures and captions). Changing the number of kept solutions changed slightly our results in terms of the % of observed bias but yield overall similar patterns for constrained and un-constrained cases. Accordingly, the following changes were made:

- Line 399-400: the bias values were changed at 0% and 50%
- Line 404-405: We added the sentence “This pattern was observed for both constrained and unconstrained systems, although the uncertainty around the median values was greater for the unconstrained cases.”
- Line 418 and 420 the observed values were changed to 40%,20% and 80%
- Line 423-427 we added the following statement: “A similar pattern was observed, but even at high λT, the median bias remained at approximately 20% rather than dropping to 0%. Additionally, the unconstrained case exhibited greater uncertainty in the bias, especially at high λT values. In constrained cases, when λT > 4, both the median and quartiles equalled 0%, whereas in unconstrained cases, the median bias was 20%, with quartile values ranging between 10% and 40%.
- Line 438: the observed bias changed to 100%
- Line 437-439: we added a part to make the sentence clearer:” Specifically, under dynamic source conditions, the median bias in the SMM could reach 100% under low λT conditions, whereas it remained at 0% with the same initial value under constant source conditions. “
- Line 486 the estimated median bias (median added) ranges now from 60% to 0% instead of 50% to 0.
- We removed line 636 from discussion “However, in un-constrained cases, only minor reductions in bias were observed when using SMMΔ compared to SMM. The lack of constraints introduces uncertainty in contribution estimations, resulting in persistently high bias regardless of the model applied.” as when keeping several solutions and studying repartition of solutions instead of one made this statement false

The community standard in the analyses of isotope mixing is utilizing the Bayesian models that

ensure more efficient convergence and can reliably describe the correlation between mixing

fractions (authors discuss this in the last paragraph of the article). Moreover, there is a type of

existing Bayesian models that would serve as an excellent starting point for the kind of analysis

that was attempted in this study - the Markov Chain Monte Carlo algorithm with covariates.

Currently, there exists an implementation in R - MixSIAR (Stock et al. PeerJ, 6:e5096, 2018. doi:

10.7717/peerj.5096). A similar analysis may also be carried out using the FRAME model

implemented in Python (Lewicki et al., PLOS ONE, 17(11), e0277204), where the user may

define a custom mixing model equation with an arbitrary number of parameters.

Thank you for your suggestion. We agree that Bayesian models are increasingly popular due to their ability to efficiently account for various sources of uncertainty, such as data, correlations, and parameter estimates. However, the limitations of the mixing model itself (i.e., the equations used to describe the process) remain the same, regardless of whether a frequentist or Bayesian approach is employed. Since our study focuses on the inherent biases and limits of the models (rather than the application of the models themselves), the choice between a frequentist or Bayesian approach should not impact the results observed.

We also acknowledge that the strength of Bayesian models lies in their ability to account for uncertainties that we chose not to address in this study. As noted by Reviewer 2, we see the potential of the Bayesian approach for creating more user-friendly and robust mixing models. We are currently developing a Bayesian framework for dynamic mixing models. Our aim with this paper is to raise awareness of the dynamic nature of many ecological systems and to provide a tool that can estimate or assess the potential bias in different field studies.

However, the existing environments mentioned, such as FRAME and MixSIAR, are stable models designed to handle relatively fixed mixing equations. While it is possible to incorporate covariates linked to time in these models, they are not capable of incorporating λ, which is a crucial component of this study. This is why we chose not to use these platforms for our analysis.

To clarify we :

- We added explanation about frequentist VS Bayesian approach and why we cannot use already existing applications in the introduction (lines 93 to 105). (See also our response for reviewer 1 comments)
- We added the following sentence in materials and methods lines 127-134: “Because no existing Bayesian MM supports a dynamic mixing model (DMM), all modelling in this study was conducted using a frequentist approach, which is easier to implement and more widely accessible. Although the frequentist approach does not offer the flexibility and uncertainty quantification of Bayesian methods, it still provides a robust framework for comparing static and dynamic mixing models [13]. A Bayesian approach would have allowed for more sophisticated handling of model uncertainty. However, despite these advantages, the frequentist method was sufficient for the purposes of comparing the two modelling approaches and testing the hypothesis of dynamic versus static isotopic incorporation.”
- We added a sentence in materials and methods precising on what part of bias we were working on line 234-235 “For this study, all the data and parameters were considered "certain," meaning that no uncertainty was introduced into the analysis.”
- We modified and refined the paragraph discussing Bayesian in discussion to highlight more the challenges and benefits around this question. Line 715-737: “However, the DMM cannot be directly integrated into existing models such as SIAR [18], MIXSIAR, or FRAME [41], as these are static models that rely on the static mixing equation (Eq. 1). Although FRAME allows for alternative mixing equations, it does not account for temporal variation in sources and consumer signatures. Some attempts have been made to incorporate temporal effects in MixSIAR by adding temporal variability in source signatures as a residual error factor [42], making it effectively an SMMΔ. Alternatively, the model can be run separately for each sampling period, creating a series of SMMs. However, neither of these approaches captures the temporal dynamics of the consumer. This raises the possibility of a Bayesian DMM implementation. The existing Bayesian mixing models coded in R use the JAGS language to run Markov chain Monte Carlo (MCMC) simulations, but JAGS does not support differential equation resolution, which is essential for the DMM (Eq. 5). A possible alternative is to implement MCMC using the Stan language, which performs similarly to JAGS while supporting differential equations.

A Bayesian DMM would offer the same diet estimation performance as the current DMM but with significant improvements in handling uncertainty. This approach allows residual error modelling without involving temporal patterns and accounts for errors related to model assumptions such as predation mode, which may require alternative mixing equations [41, 42]. The novelty of a Bayesian DMM over traditional Bayesian mixing models lies in its temporal approach, which enables the integration of isotopic data. This would introduce a new parameter: λ. Furthermore, Bayesian implementation would provide flexibility in defining λ values, allowing for the use of informative or noninformative priors based on available data quality, ultimately improving parameter estimation and uncertainty assessment.”

Bias depending on diet switch (lines 376-388) The effects of the diet switch discussed in this section depend trivially on the initial conditions of designed in-silico experiments.

“maximum bias caused by the switch effect is influenced by the Euclidean distance between the

consumer's signature prior to the diet switch and its equilibrium state“

This is trivial, similarly to the different behaviour for diets 1, 2 and 3 as explained below.

We showed in Appendix 2 (Appendix 7 on the previous draft) that the isotopic-space size in itself had no effect on bias. What has an effect is the relative movement of the consumer within this polygon. To bring a clearer illustration of that, we standardized the Euclidian distance with the “% of Switch” which is the Euclidian distance between the consumer and equilibrium divided by the isotopic space. Then, the Switch effect is the effect of this relative distance to equilibrium and it is what is studied in the first experiment (Fig 4 and 5)

-Line 255-257: We introduced the switch as “The Euclidean distance between the initial signature and equilibrium, divided by the longest distance in the source polygon, is referred to as the "switch" and is expressed as a percentage.”

- Line 311-312: The Euclidian distance was changed by: “The switch percentages were also calculated.”

- line 398-399: The Euclidian distance was changed by: “the bias increases with the percentage of switch”.

- Lines 415-417: the Euclidian distance and its values were changed by: “the maximum bias caused by the switch effect was influenced by the switch percentage (distance of the initial consumer signature from equilibrium relative to the isotopic space). In Fig. 5, where the maximum distances to equilibrium were 40% and 60% of the switch”

“In the third diet scenario (Fig. 5B), the system is unconstrained, involving four food sources for

two isotopes. Here, no clear relationship between the distance to equilibrium and the switch

effect is observed.“ That’s because the spread of the distance to equilibrium is much narrower! The distances reach up to about 11 in diet 1, 8 in diet 2 and close to 7 (guessing from the plot) in diet 3. This is a feature of the initial conditions and the lack of a clear relationship is its trivial consequence. The conclusion is misleading - as if depending on a diet a different behaviour of diet switch effect is

to be observed, which is definitely not true.

“Unlike the constrained scenarios (Fig. 4, Fig. 5A), there is no consistent reduction in bias with

λT. In this case, the bias remains elevated until the λT value reaches 5, after which it rapidly

declines to 0 when λT> 5.”

This statement, while not wrong, is also misleading. The observed dependence on λT is once

again a trivial consequence of initial conditions. What we have in reality, is the 2-dimensional

phase space in λT and Euclidean distance and the ‘bias’ is the result of model calculation for a

given pair of λT and distance. The quoted statement, as well as the previous one, is a

consequence of the uneven coverage of the λT-distance phase space across the 3 different

diets and nothing more. In fact, in terms of visualization of the results, a much clearer message

could be conveyed if λT and distance were denoted on the axes and the bias (as a result)

shown with a colour scale or similar. This way, also the uneven coverage of λT-distance phase

space would become visible immediately.

This statement does not exist anymore as by keeping several solutions for models the patterns are observed in unconstrained systems as well.

As for the λT dependence, as stated in the previous comment, it does depend of the initial conditions which is the “switch effect” that we try to observe in these experiments.

In-silico experiment with sinusoidal dependence on time of

consumer signatures

(lines 302-321)

The preparation of the experiment raises a number of questions requiring further clarification.

Why even consider 3 sources if one of the contributions is set at zero? This creates an

unnecessary confusion.

The third source was implemented to generate 2 isotopes and 3 sources environment study case because it is a complex system that usually resemble system studied in diet estimation articles. Furthermore, even if the real diet contribution of this source is 0, it is still integrated in the model inputs as a potential food source and the model will sometime attribute it a non-null estimation of participation to the diet generating bias in estimations. As in field work study, all potential food-source are integrated to the inputs even though they don’t actually participate in the diet.

Further, it is not clear how (and why) Eq. 5 was used to generate the consumer isotopic

signatures. Was it solved analytically in the form of 𝑑𝛿𝑋/𝑑𝑡 = 𝜆(𝛿𝑋𝑒𝑞(𝑡) − 𝛿𝑋), where

𝛿𝑋𝑒𝑞(𝑡)=∑𝑞𝑖(𝑡)𝛿𝑋i(𝑡) and 𝜆=const? If a numerical solver was used, then what was the number of

free parameters? As I understand, both 𝑞𝑖(𝑡) and 𝛿𝑋i(𝑡) are given, so the 𝛿𝑋(𝑡) can be calculated

directly from Eq.3 - why then not use it?

Moreover, it is not clear how Eq. 5 was used to obtain a solution for the randomly sampled

dates.

This equation can only be used if 𝛿𝑋𝑒𝑞 and λ are fixed values. In a case when the source signatures are variable over time (which is the case here), then 𝛿𝑋𝑒𝑞 changes along time and is not fixed. In this case the time model (eq.3) cannot be used to describe the evolution of 𝛿𝑋(𝑡), which is why we must use eq. 5.

We realized that the functioning of the DMM using (eq.5) was not really clear so we rephrased the materials and methods part explaining the functioning of DMM.

-line 161 changed the sentence “The third model is the DMM, which takes a different and dynamic approach.” By “The third model takes a different approach.”. For context we added “In studies examining diet switches,” to the next sentence.

-line 167-169: We added the sentence: “This model is applicable when δXeq and λ are constant over time.” We also added “However, in the DMM, the assumption is that…” to the next one.

-line 174-185: we rephrased this paragraph as: “The DMM uses an ordinary differential equation version of the time model (Eq. 5) [13, 34]. Equation (3) provides an analytical solution to Equation (5) when no components vary over time. However, when source signatures or λ change over time, no analytical solution of Equation (3) exists, and Equation (5) is used to model how the consumer’s isotopic signature changes (dδX/dt, Eq. 5). To estimate the consumer signature at each time point (δX(t)) during the period of interest, a numerical solver from the deSolve (ver 1.40,[48]) package is used. This method allows for easy incorporation of changes in both source signatures and λ over time.

Line 327 reads that “the same model parameters as the environmental setup” were used

- does it mean that the 𝛿𝑋i(𝑡) and 𝜆=const were treated as known parameters and the 𝑞𝑖(𝑡) was

obtained using the numerical solver? Then again, my question is why Eq. 3 was not used

instead?

Line 363-364 we added at which model parameters we were referring: “(same $q_{i}$, precision, TDF and λ)”.

325-326: “with linear interpolation applied between two sampled points to approximate the

source dynamic”

This approximation is not justified for larger time intervals. Perhaps, it was the authors’ intention

to show that there is no gain from using DMM in such cases, but then the discussion in lines

415-425 does not reflect that. Any considerations for λT>2 should be trivially dismissed - the

linear approximation of time dependence renders the DMM results useless for longer time

intervals between the samples.

The authors fully agree with this statement. Sampling over extended time periods is not appropriate for studying diet dynamics, and this is true for all three models considered in this study. We have addressed this point in lines 550-555 of the manuscript. To make this clearer, we have added the following sentences in the discussion (lines 553-555): “Estimating a diet with λT > 2 is risky for any model (SMMΔ or DMM), and if SMMΔ is used, the optimal sampling time corresponds to λT ~ 2 (see Fig. 10).

Geese case study

355-358

There is no illustration of the available data in the δ13𝐶 vs δ15𝑁. The results in Fig. 9 are difficult

to interpret without this kind of plot.

Biplots illustrating δ13𝐶 vs δ15𝑁 values were added to appendix 5 showing all data and additional results.

Line 381-382 we changed the sentence by: “Further details of the dataset and additional data illustrations can be found in the S5 Appendix.

“λT value (which is the time in days between two samplings multiplied by the turnover rate)”

This is a mistake - the T is the time elapsed from the diet switch. The sampling frequency is

uncorrelated with the time of the diet switch.

“We hypothesized that the DMM results closely approximated the geese’s real

diet and used these results as a reference to evaluate potential biases in the SMM estimates.”

The obtained dependence of bias vs λT (Fig. 9) is a direct consequence of the wrong

assumption discussed above. It was demonstrated earlier in the manuscript that the differences

between DMM and SMM vanish for high λT so that part of the plot is not surprising. However, at

small λT, the results are biased towards 𝛿𝑋(0) due to overestimated 𝑒−𝜆𝑡 factor (Eq. 3).

Considering the above, I cannot agree with the presented observations.

The DMM estimates the mean diet between two samplings. That means that each time it is used, it considers the first sampling as t=0 after a switch to a new diet (the one estimated) and the second sampling as the end of the experiment. So, in this case, the time T elapsed from a diet switch is equivalent of the sampling frequency. However, this comment shows that the description of the DMM functioning remains unclear so we added lines 188-193 to the materials and methods part: “The DMM is used to estimate the average diet of a consumer between two sampling dates, i.e., over a time window (T). The first sampling date is set as t = 0, marking the start of T (and any possible change in diet from a previous one), whereas the second sampling date represents the end of T. For longer time series with more than two sampling dates, the DMM is applied between each pair of sampling dates (i.e., each time window) to estimate the mean diet at each T.”

Discussion and Conclusions

I refrain from commenting on the discussion and conclusions in the final sections of the

manuscript. The authors aim to demonstrate the shortcomings of static models compared to the

dynamic ones, however, the reasoning is based on flawed methodology and thus a meaningful

discussion can only be held once the fundamental analytical flaws and weaknesses in the

design are ruled out first.

We acknowledge the reviewer’s concern, but we respectfully disagree with the assessment that the methodology is fundamentally flawed. We have thoroughly addressed the comments and recommendations provided by Reviewer 1 and 2, which led to significant improvements in the analysis, including the implementation of a more robust bias calculation method. Despite these adjustments, our results remain consistent, supporting the conclusions regarding the advantages of dynamic models over static ones.

The modifications made were carefully considered and have strengthened the robustness of our findings. As the concerns regarding the methodology have been addressed and our results hold steady, we believe the discussion and conclusions are now well-supported by the revised analysis.

We hope this clarifies our approach and reassures the reviewer of the validity of our conclusions.

Other remarks

● 41: “The analysis of stable isotope is a widely used technique in ecology”

Too wide of a statement - did you mean the modelling of stable isotope mixing? Or more

generally, the studies of isotopic composition of tissues?

Statement changed line 39 by “The study of isotopic composition in tissues”.

● 41-42: Both sentences would benefit from adding citations of relevant review literature.

- Line 39 added a citation from a review about the use of stable isotopes in trophic ecology.

Boecklen WJ, Yarnes CT, Cook BA, James AC. On the use of stable isotopes in trophic ecology. Annual review of ecology, evolution, and systematics. 2011;42(1):411-40.

-Line 40: Added 5 citations illustrating the statement “This study provides insights into trophic levels and food web structure [2-6], as well as the origin of carbon in trophic webs [4].”

Giraldo C, Ernande B, Cresson P, Kopp D, Cachera M, Travers‐Trolet M, et al. Depth gradient in the resource use of a fish community from a semi‐enclosed sea. Limnology and Oceanography. 2017;62(5):2213-26.

Medina-Contreras D, Arenas-González F, Cantera-Kintz J, Sánchez-González A, Giraldo A. Food web structure and isotopic niche in a fringe macro-tidal mangrove system, Tropical Eastern Pacific. Hydrobiologia. 2020;847:3185-99.

Fry B. Stable isotope ecology: Springer; 2006.

Baltensperger AP, Huettmann F, Hagelin JC, Welker JM. Quantifying trophic niche spaces of small mammals using stable isotopes (δ15N and δ13C) at two scales across Alaska. Canadian Journal of Zoology. 2015;93(7):579-88.

Jankowska E, De Troch M, Michel LN, Lepoint G, Włodarska-Kowalczuk M. Modification of benthic food web structure by recovering seagrass meadows, as revealed by trophic markers and mixing models. Ecological Indicators. 2018;90:28-37.

● 60-63: While the isotopic turnover rate is indeed missing in mixing models, it is not

“overlooked”, but rather the authors assume that the incorporation time window is short

enough in the context of their studies. Please remove the statement or reformulate it.

Statement changed line 61-62 by “has received much less attention in diet studies”. We cannot know about authors assumptions as it is not clearly written in articles. cf Appendix 1: the concept of turnover is not even mentioned in 74% of the reviewed articles.

● 63-64: “Other aspects of isotopic systems are also bound to time dynamics, such as

sources and consumer signatures”

This statement is not precise enough. Please name explicitly what other processes may

affect the isotopic signatures of sources and consumer tissues.

Sentence changed line 63-64 by: “Both source and consumer signatures are subject to temporal dynamics,”

● 64-65: “observed and demonstrated”

It is not clear from the context what is the difference between an observation and the

demonstration.

We added the following precisions lines 64-65 and separated the citations: “observed in field data [20] and demonstrated in theoretical or in silico models [13,21-23].

● 63-67: The discussion of potential errors due to the steady state hypothesis is very

important in the context of this article. I would be interested in a more elaborate

description of observed biases in [8,15-18] (magnitude, statistical significance, influence

on the conclusions).

We added line 66-67 the following sentence: “Ballutaud et al. [13] reported that diet estimation errors in a two-source system can reach 50%.”

● 69-71: “accounting for source variability by measuring the sources’ signatures over a

time-window relative to the isotopic incorporation before measuring the consumer

signatures”

I cannot understand this sentence.

The sentence was changed by the next one line 70-72: “This model accounts for source temporal variability by averaging the sources’ signatures over a time-window relative to the isotopic incorporation before measuring the consumer signatures in order to reduce the estimation bias.”

And in consequence, I also do not understand what

is meant by “this disparity” in the next sentence.

We added line 72-73: “the disparity in the treatment of TDF and λ”.

Editorial remarks

(only for the introductory section)

● 16: “based on the hypothesis of stable isotopic systems remains stable over time”. ->

verb problem (remaining), repetition of “stable”

Changed with reviewer 1 comment. The sentence is now line 15-16 “Most of these models assume that stable isotopic systems remain stable over time”

● 17: “referred” -> “referred to”

done

● 18: “ —” remove space

done

● 23-24: “this study aims to (...) developing a dynamic mixing model capable of

accounting for isotopic dynamics” -> verb problem

corrected

● 39: “A time window” -> article “a” not needed

corrected

“Concentration in element on source” -> “in” and “on” seem to be misused.

corrected

“Contribution of source i to diet” -> I would explicitly write “fraction” to avoid ambiguity

Changed by “Dietary contribution”

● 44: “(referred also to Table 1 for acronyms and notations)” -> grammar problems

corrected

● 46: “difference with the mixing” -> with respect to

Line 44-45 sentence changed by “the “the "plus a few ‰" difference between the consumer’s isotopic signature and the signatures of its food sources”

● 50: The phrase "when both equilibrate" is somewhat ambiguous. It could be clarified to

specify that it refers to the isotopic signature in the consumer's tissues reaching a stable

state after consuming the diet over time.

The sentence was changed line 51-52 in order to be clearer: “Second, there is a delay (or incorporation time window) between the onset of a new diet and the consumer’s full tissue equilibrium, which depends on the isotopic turnover rate (λ).”

● 52: “time lag” sounds too informal.

Changed by line 51 “delay”

● 54-55: “λ (...) can be also expressed as a function of the half-life” -> then, the inline

equation should rather be: λ = log(2)/t1/2.

corrected

● 56: “using a temporal setting” -> The statement is too vague.

Changed by line 55 “temporal approach”

● 59: “dynamic” -> dynamics?

done

● 61: “λ seems somehow overlooked in diet studies“ -> too informal

Changed by line 61-62 “λ seems to receive much less attention”

● 66 and 68: repeated phrase “these errors”

Corrected

Editor reviews:

**Dear Authors,**

After a thorough evaluation of your manuscript "From Static to Dynamic: Embracing dynamics in Isotopic Diet Estimation", I must inform you that while the study addresses a relevant issue within stable isotope mixing model research, it requires **substantial revisions** before it can be considered for publication. The manuscript has been subjected to rigorous peer review, and both reviewers have provided detailed critiques highlighting **critical methodological flaws, issues with data interpretation, and deficiencies in manuscript clarity and structure**. To be considered for further review, the manuscript must undergo a **comprehensive revision that fully addresses all major concerns**.

Failure to address these methodological concerns will render the manuscript unsuitable for further consideration.

The **most critical concern** pertains to the **methodology**, specifically the calculation of bias and the implementation of mixing models. The definition of bias used in the manuscript is fundamentally flawed, as it results in **a trivial summation to zero**, potentially masking meaningful deviations in the data. The revised version must instead adopt an approach that assesses **individual source biases**, allowing for a clearer and statistically valid interpretation of model performance. Additionally, the manuscript **fails to account for probability distributions**, which is crucial when discussing bias in an underdetermined system. The revised analysis **must incorporate these distributions**, rather than relying solely on central values such as means or medians.

The methodology flaws were identified following reviewer 2 comments and addressed. The bias is now estimated by summing the absolute value of individual source bias that avoid the summation to zero (see reviewer 2 comments and answers line 544-591).

Similarly, the issue with probability distribution was addressed by keeping several solutions to the model and studying the distribution of results instead of looking only to the best solution. It also helped us get clearer results about the observed patterns (see reviewer 2 comments and answers line 605-688).

An **equally pressing issue** concerns the **implementation of mixing models**. The current brute-force enumeration disregards the **inherent correlations between sources**, significantly weakening the validity of the findings. Given that Bayesian models are the **established standard** for handling such complexities in stable isotope research, your manuscript **must integrate Bayesian methods**, either through direct implementation or via a comparative analysis that explicitly demonstrates the limitations of brute-force approaches. The revised manuscript must include a clear discussion of **MixSIAR (Stock et al., 2018) and FRAME (Lewicki et al., 2022) models**, as well as an improved justification for the chosen methodological framework.

Source correlation appear when the model fails to discriminate between different sources. While this study does not study correlation, the experiments were set in a way that every source is discriminated. Also, as we study relative bias of the entire system using individual source bias, any correlations are incorporated within the bias and has not impact on it and our results. In the case study, as stated in the manuscript, two sources were joined ̶ as per Philips et al.,2014 recommendations ̶ as they presented a correlation. See comments from reviewer 2 line 605-625.

Bayesian methods are indeed standard in the field but unfortunately there are no existing framework that would allow us to implement the dynamic mixing model. This is because this model uses a differential equation that incorporates the isotopic turnover that is different from the existing models such as MixSiar and Frame. The justification on the choice of approach and why we do not use Bayseian approach is now discussed thoroughly in the manuscript and in reviewer 1 and 2 answers. The manuscript also now integrates a clear discussion of MixSIAR and FRAME models. These questions are addressed line 55-73 and 689-757.

Beyond these methodological issues, the **structure of the manuscript requires substantial reorganisation**. The discussion is overly long and repetitive, often restating results rather than providing a concise synthesis of their implications. The revised version must **eliminate redundant sections, improve coherence, and ensure that findings are positioned within the broader context of stable isotope research**. Additionally, the introduction **overgeneralises prior work**, suggesting that most studies fail to account for temporal dynamics. This statement is **too strong and must be rephrased**, as it currently lacks sufficient justification and risks misrepresenting the field.

The structure was strongly restudied especially the discussion part (see various comments of reviewer 1). Additional context was also added in discussion (reviewer 1 line 304-365) and more citations from literature were added to ensure a better contextualization (see reviewer 1 and 2 comments). The number of citations was 61 in the previous version of this manuscript and is now 83.

The introduction was modified and rephrased to answer for reviewer 1 and 2 comments. The statement about studies not accounting for temporal dynamics was softened see comment line 518-543.

The **interpretation of results** also requires significant improvement. The analysis of **bias dependency on diet switches** appears to be **driven by artefacts of the experimental setup rather than biologically meaningful patterns**. It is imperative to clarify **which observed effects stem from methodological design choices and which reflect genuine ecological dynamics**. Similarly, the **case study on geese is incomplete**, as it lacks a **visual representation of the δ¹³C vs δ¹⁵N data**, making it difficult to evaluate the model outputs. This **visualisation must be included**, along with a **revised explanation of λT**, which is currently misdefined by conflating turnover rates with sampling frequency.

We showed In Appendix 2 (Appendix 7 in the previous manuscript version) that the bias dependency of diet switch was independent of the experimental setup and was then an observed pattern. Following reviewer 2 comment, we cleared this reference to the appendix and clarified the materials and Methods part so it would be clearer (see reviewer 2 comments line 758-778).

Also, following reviewer 2 comment, we added visual representation of the geese data in Appendix 5 so it would give a clearer idea of the data. (line 858-865)

Similarly, following the comment of reviewer 2, we realized that the explanation of λT was unclear as it actually is the combination of turnover rate and sampling frequency in the case study. We modified the materials and Method part explaining that (see reviewer 2 comment line 866-885)

All of these questions were addressed in the answer to reviewer 2 comments.

In addition to these substantive concerns, the manuscript requires **a major revision of its language and presentation**. The text contains **unclear phrasing, excessive technical jargon, and numerous grammatical inconsistencies**, all of which hinder readability. A **thorough linguistic revision** is necessary to meet publication standards. Furthermore, the **figures require extensive revision**—captions must be self-explanatory, and visual representations must be refined to more clearly illustrate key patterns. Where needed, **new figures should be generated** to better convey complex relationships, particularly in the bias analysis across different diet scenarios.

As written in reviewer 2 comment, we passed our study through The Springer Nature Author Services for the Silver Language Editing service to improve grammatical and language issues. The certificate can be found attached or using the following code in the SNAS website: 5891-6FC4-78A8-FBFF-C759. (line 511-514)

Answering for reviewer 1 comment, every figure caption was revised (line 94-196). Also, following reviewer 2 comments, figures 4-9 were redone as well as appendix 2,5 and 6 to better convey the results (line 589-591).

Given the extent of necessary revisions, **a resubmission will only be considered if all major concerns are fully addressed**. The revised manuscript **must include a detailed response letter** outlining precisely how each of the reviewers’ critiques has been incorporated. **Failure to adequately resolve these fundamental methodological and interpretative issues will result in the manuscript being deemed unsuitable for further consideration.**

I strongly encourage you to approach these revisions with the depth and rigour that this subject matter demands. If these fundamental concerns are **comprehensively addressed**, the study has the potential to make a **meaningful contribution to the field**. I look forward to receiving a **significantly improved** manuscript in due course.

Other changes in the manuscript:

- line 536-539 we removed as not relevant anymore: “Furthermore, in cases when the source polygon is notably wide (Euclidian distance between two sources > 11.1, see S7 Appendix) the maximal bias at low λT values is higher than in typical systems. Conversely, when the consumer exhibits dynamic 515 behavior, a DMM must be employed to ensure unbiased estimations.”

- Added the mention to the Mass model line 586.

-Line 414: Modified the sentence: “When the reference diets were compared”

- The conclusion part was renamed line 685: “Conclusion and further perspectives”

-Line 40: We added a line to our table: “T	Time window between two samplings (d)”.

- We realized that our Geese dataset had switched temporal values compared to the original and corrected it. We changed Appendix 5 presenting the dataset along with the Figure 9 and the text line 476-491 describing the result which is now: “Notable differences emerged between the results of the two models (SMM and DMM; Fig. 9A, B). The first distinction lies in their approach to diet estimation. The SMM estimates the diet at each individual sampling point, whereas the DMM operates over time windows, requiring data from at least two sampling points to estimate the diet. For example, the estimation at 92 days reflected the mean diet from Day 0 to Day 92. Second, differences appeared in the estimated contributions of each source. For example, on Day 123, the SMM estimated a diet primarily composed of Grass and Ulva and Enteromorpha, whereas the DMM estimated a diet including grass, Zostera, Ulva, and Enteromorpha, with a majority of Grass. Visually, the period with the greatest difference occurred between Days 400 and 500. These disparities were also reflected in the estimated median bias (Fig. 9C). The two highest bias estimates were observed at the sampling points on Days 457 and 123. The estimated median bias ranged from 0–35% across λT values ranging from 1.11– to 9.7. Here, the λT value is derived from the period between two sampling points, varying from 31 days (λT = 1.11) to 273 days (λT = 9.75), assuming a constant λ throughout the study. Consistent with theoretical predictions under constant isotopic source values for both pattern and observed values, the estimated bias decreased as λT increased, becoming negligible when λT > 4.”
