# Supplementary material for: From static to dynamic: Embracing dynamics in isotopic diet estimation
Source: PLoS One. 2025 Aug 26;20(8):e0330327. doi: 10.1371/journal.pone.0330327 (PMC12380277; doi:10.1371/journal.pone.0330327)
Supplement: Attachment — Submitted filename: Response_to_Reviewers_auresp_2.docx [file pone.0330327.s010.docx]

**From Static to Dynamic: Embracing Dynamics in Isotopic Diet Estimation**

Emilie Cathelin^1*^, Sebastien Lefebvre^1^, Carolina Giraldo^2^

This is the answers to the Editor comments. Hereafter in the document, editor’s comments will appear in black while the answers will appear in blue. Text appearing underlined was added to the manuscript.

Dear Dr Cathelin and co-authors,

I have now carefully reviewed your revised manuscript entitled “From Static to Dynamic: Embracing dynamics in Isotopic Diet Estimation”, together with your detailed responses to both the reviewers’ and my editorial comments. I appreciate the substantial improvements you have made. Most of the key concerns from Reviewer 1 and my own editorial suggestions have been addressed thoroughly, and the manuscript is much clearer in its current form.

However, I note that several fundamental methodological criticisms raised by Reviewer 2 — particularly regarding the neglect of source correlations and the absence of uncertainty quantification — have not been fully addressed in the current version. While you have justified your choice of a deterministic framework and the lack of existing Bayesian dynamic models, these limitations must be presented very clearly to ensure transparency and to avoid overgeneralising the practical recommendations for real-world applications.

Therefore, I am inviting you to submit a revised version of the manuscript addressing the following specific points:

Explicitly acknowledge in the Discussion and Conclusions that your approach does not model uncertainty or correlation structures, unlike standard Bayesian mixing models. Please clarify that your results are based on central estimates only and that this may limit the interpretation of bias magnitude.

We thank the editor for the positive feedback and the opportunity to further clarify the methodological scope of our study. In response to the remaining concerns regarding source correlations and uncertainty quantification, we have made the following changes to ensure full transparency and to better guide readers in interpreting the results.

We first added a sentence to the materials and methods section (lines 234-239) stating that the model do not neither deal with error structure nor correlation diagnosis: “The code provides functions to running all three mixing models using a deterministic approach, as well as several graphical outputs to ease interpretation of the results and to assess fittings. However, the model is implemented assuming no data or parameter-based uncertainty and does not address error structure arising from data uncertainty, nor does it include a formal diagnosis of contribution correlations which must be conducted separately.”

We also added a clarification to Line 135 in the materials and methods section and a reference to Stock and Semmens, 2016 article: “A Bayesian approach would have allowed for a more sophisticated treatment of model uncertainty and error structure [42].”

In the discussion, we added a paragraph highlighting the limitations of the current approach (lines 699-705):

” Another limitation of the DMM as implemented (using a frequentist approach) lies in its reliability assessment. This model assumes no data or parameter-based uncertainties, making it impossible to investigate error when applied to a dataset. Moreover, it does not assess the correlation structure within the model itself unlike standard mixing models [19]. As a result, the DMM is less user-friendly for now, providing no direct estimates of contribution correlation and yielding lower precision in error structure estimation compared with existing mixing models. These issues could be managed by adapting the DMM to a Bayesian approach**.”**

We also added a concluding statement (lines 735-736) to highlight the lack of uncertainty treatment within the current DMM: **“To further advance the DMM, adapting it to a Bayesian framework could be considered. Bayesian approaches are well suited to incorporating data uncertainty [83], which the DMM currently lacks, and are increasingly used in diet estimation, as demonstrated by the MixSIAR package [19].”**

Finally, we added in Supplementary materials (Appendix 7) a table comparing the current DMM with MixSIAR clearly showing the differences in application hypothesis, error structure, correlation etc. We added a mention to this Appendix line 744-745: “However, neither of these approaches captures the temporal dynamics of the consumer (see Appendix 7 for further details).”

Most of the result graph (Figures 4,5,9A. B) presents quartiles value along with the central value (here the mean) to represent the bias (or contribution for figure 9) magnitude. To clarify the graphical representations of uncertainty, we added the following details:

- in the Fig 6 caption (lines 459-460): “Fig 6: Effect of source variability on bias as a function of λT for three initial consumer values in diet 1 (with three sources, 100% of the diet from source 3). The median biases are represented for three consumer initial values: one close to equilibrium (A, 0% switch), one in the middle of the polygon (B, 50% switch), and one far from equilibrium (C, 100% switch). For more details, see Fig 4C. The coloured points represent the median bias value in the source-constant scenario for solutions falling within a 0.5‰ tolerance. The grey envelopes represent the maximum and minimum median biases in the dynamic source scenarios for the top 5% of the best solutions. Quartiles ranges are not shown here for the sake of clarity but can be observed in Fig 4.A.”
- In Fig 8 caption (lines 484-485): “Fig 8: Boxplot of median biases for the three models relative to the field-sampled-like dataset.(A) Applied DMM, (B) SMMΔ, and (C) SMM The observations were divided into 10 classes of λT (size 0.5). Quartile ranges are not shownhere for the sake of clarity since they may widen the bias magnitude.”
- In Fig 9 caption (lines 508-509): Fig 9: Comparison of source contribution estimates over time by the SMM and DMM in the goose dataset and associated bias estimations. Panels (A) and (B) depict the estimated contributions of each food source over time, for the SMM and DMM, respectively. The lines represent the median estimates, and the envelopes show the quartile ranges. Panel (C) displays the median bias as a function of λT, with the median DMM estimations used as the reference diet. Quartile ranges are not shown here for the sake of clarity since they may widen the bias magnitude.”

Adjust any wording in the manuscript that could imply that the dynamic mixing model is fully robust for real-world application without addressing statistical uncertainty. Recommendations should be nuanced to reflect this limitation.

The recommendations in the discussion section clarify which type of model underlying equation (dynamic vs static) is most suitable for a given dataset. The discussion focuses on the DMM in general that is a model based on a differential equation rather than a static one regardless of whether it is implemented in a deterministic or Bayesian framework. In response of your comment, we have adjusted the manuscript as follows:

- Line 541: The DMM consistently produces diet estimations with minimal bias at low λT values (λT < 2), unlike those produced by static models (without accounting for data uncertainty).
- Line 545-546: “Additional recommendations for selecting and applying mixing models regardless of whether deterministic or Bayesian approaches are used, are presented in Fig 10.”
- Line 633: “These studies, which already account for temporal dynamics, could readily adopt a DMM for more accurate dietary estimates.”

Consider adding a short statement in the Data Availability and Methods sections noting that the provided scripts support deterministic replication but do not implement uncertainty propagation or MCMC sampling.

The sentence has been added to the Data availability section: “the provided scripts support deterministic replication of the analyses but do not implement uncertainty propagation or MCMC sampling.” See also in the materials and methods section for an explicit statement of this limitation.

These clarifications are important to align the manuscript with PLOS ONE’s requirements for methodological transparency and to properly inform readers about the scope and limitations of the work.

Please submit your revised manuscript and provide a concise response letter summarising how these final points have been addressed.

Thank you once again for your valuable contribution and your constructive responses throughout the review process.

We hope that these additions sufficiently clarify the scope and limitations of our approach and meet the journal’s standards for methodological transparency.
