# Supplementary material for: From static to dynamic: Embracing dynamics in isotopic diet estimation
Source: PLoS One. 2025 Aug 26;20(8):e0330327. doi: 10.1371/journal.pone.0330327 (PMC12380277; doi:10.1371/journal.pone.0330327)
Supplement: Attachment — Submitted filename: Response_to_reviewers_auresp_3.docx [file pone.0330327.s011.docx]

**From Static to Dynamic: Embracing Dynamics in Isotopic Diet Estimation**

Emilie Cathelin^1*^, Sebastien Lefebvre^1^, Carolina Giraldo^2^

This is the answers to the Editor comments. Hereafter in the document, editor’s comments will appear in black while the answers will appear in blue.

Dear Dr Cathelin,

Thank you for your thorough and constructive revision of the manuscript entitled "From Static to Dynamic: Embracing Dynamics in Isotopic Diet Estimation".
I have carefully reviewed the revised version and I am pleased to confirm that the manuscript now meets the scientific and editorial standards required for publication in PLOS ONE. The changes you have implemented are appropriate and well executed, and your responses to the previous editorial comments are satisfactory.

However, in accordance with PLOS ONE’s data availability policy, which requires that all data and code necessary to replicate the findings be deposited in a stable, long-term public repository, I must request a final revision before acceptance. Specifically, although the code is currently available on GitHub, this platform does not guarantee permanence or version control with a persistent identifier. To ensure long-term accessibility and the reproducibility of the results reviewed by the referees, I kindly ask that you deposit the current version of the code in a DOI-minting repository such as Zenodo or an equivalent archive prior to acceptance. Please also update the Data Availability Statement in the manuscript accordingly.

Thank you for your comment. All data and codes have been put in a permanent repository (Zenodo).

We changed the access link in the manuscript line 233-234:” all the scripts used to produce the results in this study are available on Zenodo (available at <https://doi.org/10.5281/zenodo.16040315)>.”

We also updated the Data availability Statement as requested.
